# Inorganic nitrate benefits contrast-induced nephropathy after coronary angiography for acute coronary syndromes: the NITRATE-CIN trial

**DOI:** 10.1093/eurheartj/ehae100

**Published:** 2024-03-21

**Authors:** Daniel A Jones, Anne-Marie Beirne, Matthew Kelham, Lucinda Wynne, Mervyn Andiapen, Krishnaraj S Rathod, Tipparat Parakaw, Jessica Adams, Annastazia Learoyd, Kamran Khan, Thomas Godec, Paul Wright, Sotiris Antoniou, Andrew Wragg, Muhammad Yaqoob, Anthony Mathur, Amrita Ahluwalia

**Affiliations:** William Harvey Research Institute, Barts & The London Faculty of Medicine & Dentistry, Queen Mary University of London, Charterhouse Square, London EC1 M 6BQ, UK; Barts Interventional Group, Barts Heart Centre, Barts Health NHS Trust, London, UK; Barts Cardiovascular Clinical Trials Unit, Queen Mary University of London, Charterhouse Square, London EC1M 6BQ, UK; William Harvey Research Institute, Barts & The London Faculty of Medicine & Dentistry, Queen Mary University of London, Charterhouse Square, London EC1 M 6BQ, UK; Barts Interventional Group, Barts Heart Centre, Barts Health NHS Trust, London, UK; William Harvey Research Institute, Barts & The London Faculty of Medicine & Dentistry, Queen Mary University of London, Charterhouse Square, London EC1 M 6BQ, UK; Barts Interventional Group, Barts Heart Centre, Barts Health NHS Trust, London, UK; Barts Interventional Group, Barts Heart Centre, Barts Health NHS Trust, London, UK; Barts Interventional Group, Barts Heart Centre, Barts Health NHS Trust, London, UK; William Harvey Research Institute, Barts & The London Faculty of Medicine & Dentistry, Queen Mary University of London, Charterhouse Square, London EC1 M 6BQ, UK; Barts Interventional Group, Barts Heart Centre, Barts Health NHS Trust, London, UK; William Harvey Research Institute, Barts & The London Faculty of Medicine & Dentistry, Queen Mary University of London, Charterhouse Square, London EC1 M 6BQ, UK; Barts Cardiovascular Clinical Trials Unit, Queen Mary University of London, Charterhouse Square, London EC1M 6BQ, UK; Barts Cardiovascular Clinical Trials Unit, Queen Mary University of London, Charterhouse Square, London EC1M 6BQ, UK; Barts Cardiovascular Clinical Trials Unit, Queen Mary University of London, Charterhouse Square, London EC1M 6BQ, UK; Barts Cardiovascular Clinical Trials Unit, Queen Mary University of London, Charterhouse Square, London EC1M 6BQ, UK; Department of Pharmacy, Barts Heart Centre, Barts Health NHS Trust, London, UK; Department of Pharmacy, Barts Heart Centre, Barts Health NHS Trust, London, UK; William Harvey Research Institute, Barts & The London Faculty of Medicine & Dentistry, Queen Mary University of London, Charterhouse Square, London EC1 M 6BQ, UK; Barts Interventional Group, Barts Heart Centre, Barts Health NHS Trust, London, UK; William Harvey Research Institute, Barts & The London Faculty of Medicine & Dentistry, Queen Mary University of London, Charterhouse Square, London EC1 M 6BQ, UK; Department of Nephrology, Barts Health NHS Trust, London, UK; William Harvey Research Institute, Barts & The London Faculty of Medicine & Dentistry, Queen Mary University of London, Charterhouse Square, London EC1 M 6BQ, UK; Barts Interventional Group, Barts Heart Centre, Barts Health NHS Trust, London, UK; William Harvey Research Institute, Barts & The London Faculty of Medicine & Dentistry, Queen Mary University of London, Charterhouse Square, London EC1 M 6BQ, UK; Barts Cardiovascular Clinical Trials Unit, Queen Mary University of London, Charterhouse Square, London EC1M 6BQ, UK

**Keywords:** Nitrate, Nitric oxide: contrast induced nephropathy, Acute coronary syndrome, Angiography, Renoprotection

## Abstract

**Background and Aims:**

Contrast-induced nephropathy (CIN), also known as contrast-associated acute kidney injury (CA-AKI) underlies a significant proportion of the morbidity and mortality following coronary angiographic procedures in high-risk patients and remains a significant unmet need. In pre-clinical studies inorganic nitrate, which is chemically reduced *in vivo* to nitric oxide, is renoprotective but this observation is yet to be translated clinically. In this study, the efficacy of inorganic nitrate in the prevention of CIN in high-risk patients presenting with acute coronary syndromes (ACS) is reported.

**Methods:**

NITRATE-CIN is a double-blind, randomized, single-centre, placebo-controlled trial assessing efficacy of inorganic nitrate in CIN prevention in at-risk patients presenting with ACS. Patients were randomized 1:1 to once daily potassium nitrate (12 mmol) or placebo (potassium chloride) capsules for 5 days. The primary endpoint was CIN (KDIGO criteria). Secondary outcomes included kidney function [estimated glomerular filtration rate (eGFR)] at 3 months, rates of procedural myocardial infarction, and major adverse cardiac events (MACE) at 12 months. This study is registered with ClinicalTrials.gov: NCT03627130.

**Results:**

Over 3 years, 640 patients were randomized with a median follow-up of 1.0 years, 319 received inorganic nitrate with 321 received placebo. The mean age of trial participants was 71.0 years, with 73.3% male and 75.2% Caucasian; 45.9% had diabetes, 56.0% had chronic kidney disease (eGFR <60 mL/min) and the mean Mehran score of the population was 10. Inorganic nitrate treatment significantly reduced CIN rates (9.1%) vs. placebo (30.5%, *P* < .001). This difference persisted after adjustment for baseline creatinine and diabetes status (odds ratio 0.21, 95% confidence interval 0.13–0.34). Secondary outcomes were improved with inorganic nitrate, with lower rates of procedural myocardial infarction (2.7% vs. 12.5%, *P* = .003), improved 3-month renal function (between-group change in eGFR 5.17, 95% CI 2.94–7.39) and reduced 1-year MACE (9.1% vs. 18.1%, *P* = .001) vs. placebo.

**Conclusions:**

In patients at risk of renal injury undergoing coronary angiography for ACS, a short (5 day) course of once-daily inorganic nitrate reduced CIN, improved kidney outcomes at 3 months, and MACE events at 1 year compared to placebo.

## Structured Graphical Abstract

**Structured Graphical Abstract:**
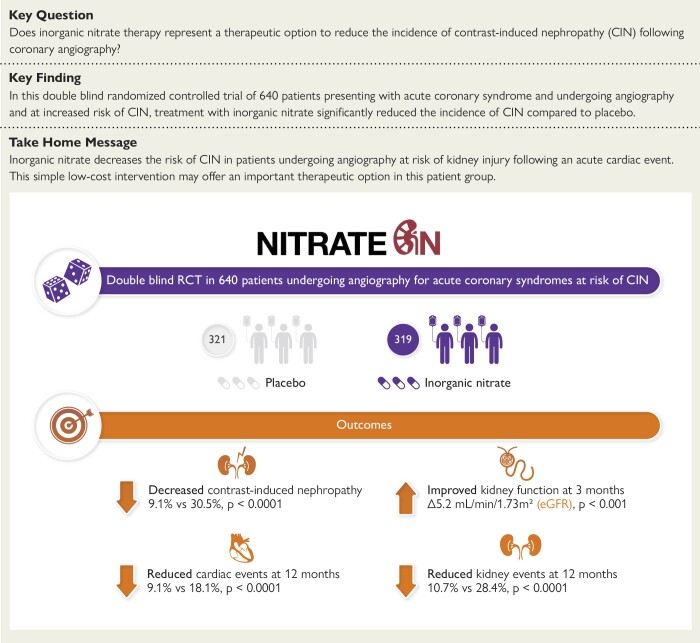
NITRATE-CIN was a double-blind randomized controlled trial (RCT) of 640 patients presenting with acute coronary syndrome and undergoing angiography and at increased risk of contrast-induced nephropathy (CIN). Treatment of patients with inorganic nitrate (12 mmol/day capsules on the day of surgery and for 4 days after) significantly reduced the incidence of CIN, improved kidney outcomes at 3 months, and major adverse cardiac events at 1 year compared to placebo. eGFR, estimated glomerular filtration rate.

**See the editorial comment for this article ‘Inorganic Nitrate: a game changer in preventing contrast-associated acute kidney injury’, by C. Zoccali, https://doi.org/10.1093/eurheartj/ehae094.**

## Introduction

Contrast-induced nephropathy (CIN) also termed contrast-associated acute kidney injury (CA-AKI), defined by a deterioration in renal function after contrast exposure, is considered a serious complication of coronary angiography.^1–3^ The incidence of CIN (CA-AKI) ranges from <1% to >50% depending on patient characteristics and comorbidities, type of procedures, and definitions used.^4,5^ CIN (CA-AKI) often occurs following coronary angiography for acute coronary syndromes (ACS) with a reported incidence of up to 55% in high-risk patients, such as those with older age, heart failure, chronic kidney disease (CKD), or diabetes with CKD.^2,6^ The clinical consequences of CIN are significant, being associated with increased risk for renal replacement therapy (RRT), longer length of hospital stay, recurrent revascularization procedures, and higher mortality.^6^

Whilst the pathophysiology of CIN (CA-AKI) remains uncertain, one of the principal mechanisms thought to underlie the condition is the release of reactive oxygen species (ROS) and vasoconstrictive renal hypoxic injury.^3,7,8^ Importantly, studies in pre-clinical models and early phase trials suggest that, in part, this oxidative stress decreases levels of protective nitric oxide (NO).^9–11^ Thus, strategies that seek to replace this ‘lost’ NO represent an approach that may confer therapeutic benefit. To date approaches to deliver NO in the form of organic nitrate have not translated well. There is some evidence that antioxidant therapy, in the form of *N*-acetylcysteine (NAC) that would indirectly elevate NO, provides some benefit,^12^ however further trials have failed to reproduce these findings.^13^

A potential alternative and more direct solution for elevating endogenous NO levels lies in targeting the non-canonical pathway for NO synthesis via the *in vivo* two-step sequential chemical reduction of inorganic nitrate (NO_3_^−^) to nitrite (NO_2_^−^) and then nitrite to NO. This activity is coupled with the major advantage over the organic nitrates and nitrites of no development of tachyphylaxis and tolerance; the characteristic that is the primary and major factor limiting use of the organic compounds in the clinical setting. Inorganic nitrate (NO_3_^−^) is used to safely and effectively enhance this non-canonical pathway,^14^ and thus we assessed the potential therapeutic benefit that could be derived from the use of inorganic nitrate in the prevention of CIN (CA-AKI) in patients with ACS.

## Methods

### Study design and oversight

NITRATE-CIN is a prospective, randomized, single-centre, double-blind placebo-controlled trial designed to test the efficacy of inorganic nitrate in patients undergoing angiography for non-ST-elevation ACS (NSTE-ACS). The trial design has been described previously.^15^ The trial was funded by Heart Research UK, via the Translational Research Project scheme and sponsored by the Queen Mary University of London. The trial was approved by an independent ethics committee (London—Surrey Borders Research Ethics Committee, reference (18/LO/1132), registered in approved registries (NCT03627130) and performed in accordance with the Declaration of Helsinki (1996) and the principles of the International Conference on Harmonization–Good Clinical Practice (ICH-GCP) Guidelines. Independent trial steering and data and safety monitoring committees oversaw the trial. The Barts Cardiovascular Clinical Trials Unit (CVCTU) oversaw the management and conduct of the trial, including case report form design, safety reporting, coordination of trial committees, statistical analysis, and database management. Data was captured in REDCap, a web-based, electronic database for all study participants, and the database was held in a secure server (Barts Safe Haven) at Queen Mary University of London. The authors had access to the trial data and vouch for the completeness and accuracy of the data.

### Study population

All consecutive patients meeting trial inclusion criteria (NSTE-ACS presentation, at risk of CIN, age ≥18, and being able to give written informed consent) who were referred for invasive coronary angiography, to be conducted as per current guidelines, to St Bartholomew’s Hospital, London, UK, were considered eligible for participation. This is the largest cardiac centre in the UK, serving a population of approximately 6 million people from Northeast and Central London and is a 24/7 centre performing approximately 6000 angiograms and 2000 non-primary angioplasties a year. Risk of CIN was defined as an estimated glomerular filtration rate (eGFR) < 60 mL/min or two of the following: diabetes, liver failure (cirrhosis), age >70 years, exposure to contrast in last 7 days, heart failure (or left ventricular ejection fraction <40%), and concomitant renally active drugs (e.g. diuretics, angiotensin receptor blockers) as per current guidelines.^2^

Patients with cardiac arrest, cardiogenic shock, or ST-elevation myocardial infarction (STEMI) were not included in the study. Patients with chronic kidney failure with an eGFR <20 mL/min and women who were pregnant were not included. Finally, patients with a current life-threatening condition other than vascular disease that may prevent the subject from completing the study were excluded.

### Randomization

Patients were randomized on a 1:1 basis to receive either inorganic nitrate or placebo. Block randomization was used with block size varied randomly and patients stratified into diabetic and non-diabetic patients. The allocation algorithm was written by the study statistician in Stata (Version 14) using a pseudorandom number generator. Treatment assignment in both the inorganic nitrate and placebo groups remained blinded until after data lock and statistical analysis at the end of the study.

### Intervention

Patients were randomized to receive two potassium nitrate capsules (KNO_3_: 6 mmol each giving 12 mmol in total equivalent to 744 mg of nitrate) or an equivalent dose of potassium chloride (KCl) control once daily for a total of 5 days with the first dose taken prior to coronary angiography. Previous studies have demonstrated the tolerability of KNO_3_ with minimal side effects.^14^ Levels of circulating NO_2_^−^ achieved with 12 mmol KNO_3_ are approximately 0.8–1.0 μmol/L 1–3 h after ingestion,^14^ which correspond well with the levels achieved following a bolus dose of sodium nitrite in NITRITE-AMI (0.67 ± 0.18 μM) that was associated with a reduction in the occurrence of CIN of 80% with nitrite treatment, identified in our post-hoc unpowered analysis of CIN rates in this cohort.^16,17^ The KNO_3_ and matching placebo (KCl) capsules were supplied by the Pharmacy Manufacturing Unit based at Guy’s and St Thomas’ NHS Foundation Trust.

### Study procedures

Blood and urine samples were collected at baseline, 4–6 h, 48–72 h, and 3 months after angiography. Baseline samples were taken before the administration of intravenous pre-hydration (see Supplementary data online, *Appendix S1*). Blood samples were immediately centrifuged for biochemical assessments. Urine was collected in Falcon tubes and stored at −80°C until analysis of nitrate and nitrite concentrations quantified using liquid phase ozone chemiluminescence as per our previous publications.^18^

### Outcomes and follow-up

The primary endpoint was incidence of CIN (≥0.3 mg/dL or ≥26.5 μmol/L increase in creatinine within 48 h or ≥1.5 × within 1 week, as defined by the Kidney Disease Improving Global Outcomes (KDIGO) criteria for acute kidney injury^19^ (full criteria in Supplementary data online, *Material*). Modification of Diet in Renal Disease-derived eGFR (mL/min/1.73 m^2^) was used in keeping with national guidance. Secondary endpoints included rates of procedural myocardial infarction (MI), kidney function measured at 3 months, levels of NO_2_^−^/NO_3_^−^ at 4–6, 48–72, and 3 months, and both major adverse cardiac events (MACE) and major adverse kidney events (MAKE) measured out to 12 months. Procedural MI was defined using the SCAI definition.^20^ MACE was defined as all-cause mortality, cardiac mortality, MI, and unscheduled revascularization. MAKE was defined as all-cause mortality, new-onset RRT, and persistent worsening kidney dysfunction (>50% increase in baseline creatinine concentration) as per current gold standard methods.^21^

### Statistical analysis

For our study calculations, we assumed a conservative CIN incidence of 12% in the control group, with a proposed reduction of 60% with inorganic nitrate. This reduction was based on preliminary data from the 80 STEMI patient NITRITE-AMI trial^17^ where an acute intra-coronary bolus of sodium nitrite (10 µmol/L) was associated with an 80% reduction in CIN compared to patients who received placebo control. 640 patients allowed a power of 80% and a significance level of 0.05, allowing for 27% dropout (primary endpoint).

All analyses were conducted on an intention-to-treat basis. The statistical analysis plan (available with the protocol) was finalized before unblinding the trial group assignments. Data are presented as mean values with standard deviations or median values with interquartile ranges. The primary outcome of CIN was analyzed using logistic regression, with estimates made unadjusted, and adjusted for diabetes status and creatinine level at baseline. Pre-specified subgroup analyses were performed on primary outcomes by incorporating and testing interaction terms into the models. Analyses of secondary outcomes were not adjusted for multiplicity. Differences in secondary outcomes between trial groups were estimated using linear regression for continuous outcomes, and logistic regression for binary outcomes with and without adjustment for diabetes status and creatinine level at baseline. Assumptions made for each model were tested and deemed not to be violated. Secondary endpoints are presented with 95% confidence intervals (CI). For the MACE and MAKE endpoint, a Kaplan–Meier plot was used to show cumulative incidence in the two treatment groups over 1-year follow-up. All analyses were conducted with the use of Stata software, version 17.0 (StataCorp., College Station, TX, USA).

### Role of the funding source

The funder (HRUK) of the study had no role in study design, data collection, data analysis, data interpretation, or writing of the report.

## Results

### Characteristics of the study population

**Figure 1:**
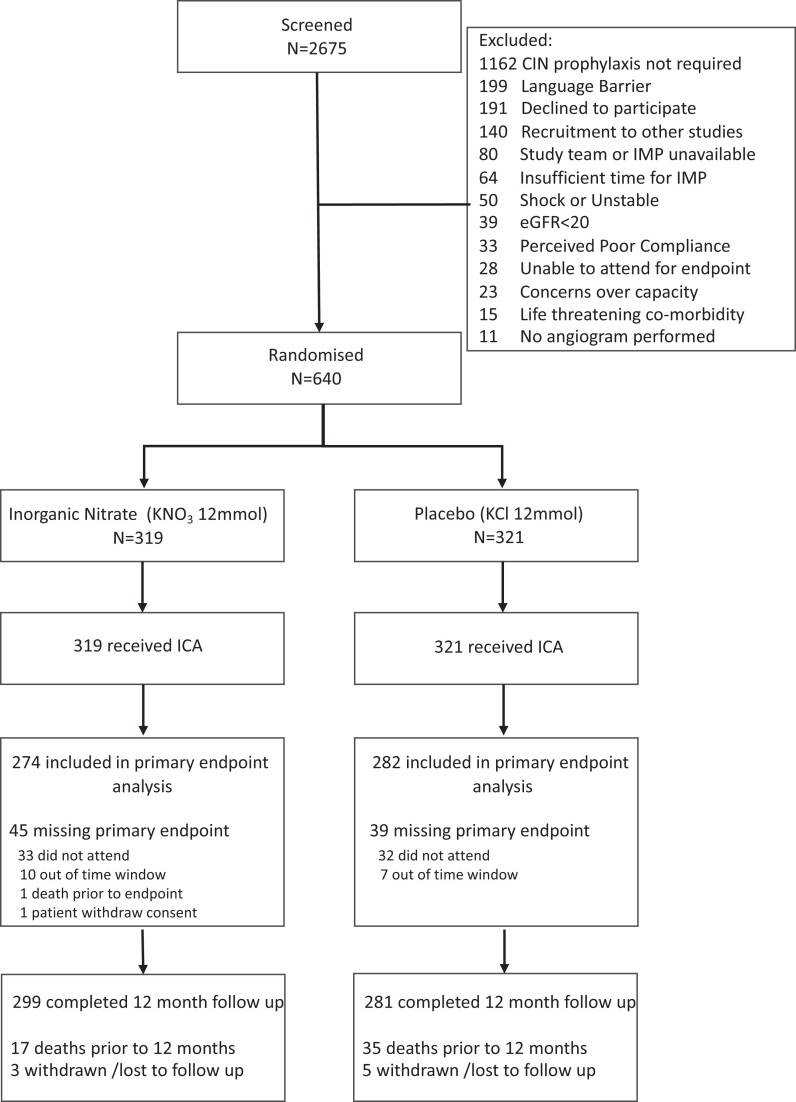
Consort diagram

Between November 2018 and July 2021, 2675 patients presenting with NSTE-ACS were referred for invasive coronary angiography at St Bartholomew’s Hospital. Among these, 1162 patients were excluded as CIN prophylaxis was not required (as per local policy (see Supplementary data online, *Appendix S1*) consistent with NICE guidelines. The remaining 1513 patients who were at risk of CIN were evaluated with the following excluded: unstable presentation (STEMI, cardiac arrest, or cardiogenic shock) (*n* = 50), eGFR <20 mL/min or established on long-term RRT (*n* = 39), study team or investigational medicinal product (IMP) being unavailable (*n* = 80), recruitment to other research studies (*n* = 140), unable to consent (language barrier, capacity) (*n* = 126) and 247 patients were felt to be unsuitable by the research team (compliance, co-morbidity, and life expectancy) so were unsuitable for randomization. This left 831 suitable patients, of which 191 declined. Data are thus presented for 640 patients (319 in the inorganic nitrate group and 321 in the placebo group) (*Figure 1*-Consort^22^ diagram).

#### Baseline demographics

The mean age of the trial participants was 71.0 years, with 73.3% male and 75.2% Caucasian (see *Table 1* for more detailed ethnicity distribution). Rates of diabetes overall (45.9%) were high, comparatively to other ACS cohorts (commonly observed at ∼25%,^23,24^ but as expected in a high CIN risk cohort. Most patients recruited presented with non-STEMI (NSTEMI) (84.8%) with similar rates of troponin-positive presentation between treatment groups (*Table 1*).

**Table 1:** Baseline characteristics of the NITRATE-CIN cohort

	Placebo (*n* = 321)	Inorganic Nitrate (*n* = 319)
**Age (years), mean ± SD**	71.2 ± 11.5	70.8 ± 11.8
**Sex, *n* (%)**		
Female	80 (24.9)	91 (28.5)
Male	241 (75.1)	228 (71.5)
**Ethnicity, *n* (%)**		
Asian	44 (13.7)	58 (18.2)
Black	31 (9.7)	22 (6.9)
White	242 (75.4)	239 (74.9)
Unknown	4 (1.3)	0
**BMI (kg/m^2^), mean ± SD**	28.6 ± 5.8	28.1 ± 5.6
**Hypertension, *n* (%)**	248 (77.3)	237 (74.3)
**Hypercholesterolaemia, *n* (%)**	190 (59.0)	196 (61.0)
**Previous PCI, *n* (%)**	105 (32.7)	107 (33.5)
**Previous MI, *n* (%)**	104 (32.4)	106 (33.2)
**Diabetes, *n* (%)**	148 (46.1)	146 (45.8)
Type I	3 (0.9)	3 (0.9)
Type II: Diet controlled,	20 (6.2)	21 (6.6)
Type II: Drug therapy,	89 (27.7)	94 (29.5)
Type II: Insulin	36 (11.2)	28 (8.8)
**Presentation, *n* (%)**		
Unstable angina	47 (14.6)	50 (15.7)
NSTEMI	274 (85.4)	269 (84.3)
**Smoking History, *n* (%)**		
Non- smoker	134 (41.7)	134 (42.0)
Previous smoker	144 (44.9)	141 (44.2)
Current smoker	43 (13.4)	44 (13.8)
**Peripheral Vascular Disease, *n* (%)**	9 (2.8)	17 (5.3)
**Stroke, *n* (%)**	21 (6.5)	17 (5.3)
**CKD (eGFR < 60 mL/min), *n* (%)**	167 (52.1)	191 (59.8)
**LV impairment, *n* (%)**	118 (37.0)	128 (40.0)
**LV Ejection Fraction, mean ± SD**	48 ± 11	48 ± 12
**SBP (mmHg), mean ± SD**	132.8 ± 19.5	130.9 ± 21.0
**DBP (mmHg), mean ± SD**	72.3 ± 10.7	71.7 ± 10.6
**HR (bpm), mean ± SD**	70.3 ± 12.6	69.6 ± 12.0

NSTEMI, Non-ST Elevation Myocardial Infarction; PCI, Percutaneous Coronary Intervention; eGFR, estimated glomerular filtration rate; SBP, systolic blood pressure; DBP, diastolic blood pressure; HR, heart rate. Kidney insufficiency is defined as eGFR <90 mL/min/1.73 m^2^.

Overall, 56.0% of patients were classified as having CKD (eGFR <60 mL/min) meeting criteria for CIN prophylaxis with comparable rates between the two groups, but again it should be acknowledged that this is higher than typical ACS cohorts reported in the literature^24–26^ due to the inclusion criteria of high risk of CIN. The remaining patients qualified for CIN prophylaxis based on the presence of diabetes (25.3%), or age over 70 (13.1%) both with accompanying nephrotoxic medication, with the remaining qualifying due to impaired left ventricular ejection fraction (<40%). Mehran scores were similar between the two groups (10.3 ± 3.4 vs. 9.7 ± 3.6) (*Table 2*). The proportion of patients qualifying in each criterion according to ethnicity is shown in Supplementary data online, *Table S1*. As per local policy (see Supplementary data online, *Appendix S1*) all patients underwent pre-hydration with intravenous fluids before angiography and Iodixanol was used as the contrast agent in all patients.

**Table 2:** Invasive coronary angiography procedural data of the NITRATE-CIN cohort

	Placebo (*n* = 321)	Inorganic nitrate (*n* = 319)
**Access route, *n* (%)**		
Femoral	45 (14)	22 (6.9)
Radial	276 (86)	297 (93)
**Contrast (mL), median [Q1–Q3]**	170 [105–235]	153 [105–225]
**Contrast >300 mL, *n*(%)**	37 (11.5)	25 (7.8)
**Radiation (mGy), median [Q1-Q3]**	248 [127–520]	211 [144–404]
**Radiation (mGy^2^), median [Q1-Q3]**	1335 [751–2346]	1221 [708–2061]
**Syntax Score, median [Q1-Q3]**	13 [8–20]	12 [7–19]
**Outcome, *n* (%)**		
Medical	128 (39.9)	137 (42.9)
PCI	160 (49.8)	147 (46.1)
Surgery	33 (10.3)	35 (10.9)
**Vessel treated, *n* (%)**		
LMS	5 (1.6)	7 (2.2)
LAD	84 (26)	66 (21)
LCx	48 (15)	39 (12)
RCA	52 (16)	51 (16)
**Intravascular imaging, *n* (%)**		
IVUS	58 (18)	49 (15)
OCT	22 (6.9)	9 (2.8)
**Pressure wire, *n* (%)**	14 (4.4)	22 (6.9)
**Adjunctive techniques, *n* (%)**		
Rotational Atherectomy	11 (6.8)	9 (6.1)
IVL	3 (2.0)	0 (0)
**Mehran score, mean ± SD**	9.7 ± 3.6	10.3 ± 3.4
**Mehran risk group, *n* (%)**		
Low (≤5)	26 (8.1)	25 (7.8)
Medium (6–10)	183 (57.0)	153 (48.0)
High (11–15)	88 (27.4)	112 (35.1)
Very high (≥16)	24 (7.5)	29 (9.1)

Data is shown as median [Q1–Q3] or mean value ± standard deviation (SD) or a number (percentage). PCI, Percutaneous Coronary Intervention; IVL, Intravascular Lithotripsy; OCT, Optical Coherence Tomography; LMS, Left Main Stem; LAD, Left Anterior Descending; LCx, Left Circumflex; RCA, Right Coronary Artery.

#### Angiographic information

Radial access rates were comparable between treatment groups (placebo vs. inorganic nitrate: 86% vs. 93%, *P* = .23) with similar quantities of contrast administered in the two groups (181 ± 95 vs. 169 ± 85 mL, *P* = .10, Supplementary data online, *Figure S1*). Most patients received percutaneous coronary intervention (PCI) in both groups (49.8% vs. 46.1%) with similar rates of patients treated with coronary artery bypass grafting (CABG) (*Table 2*).

### Primary endpoint

#### Contrast-induced nephropathy

Data for assessment of CIN were available for 556 patients of the 640 total. Of these 556 patients, 111 experienced CIN giving an overall rate of 20.0%. The majority of cases were stage 1 AKI (91.9%), followed by stage 2 (7.2%) and then stage 3 (0.9%).

The proportion of patients with CIN was significantly reduced in those treated with inorganic nitrate (9.1%) compared to the placebo group (30.5%, *P* < .001). This difference persisted after adjustment for baseline creatinine and diabetes status (odds ratio 0.21, 95% CI 0.13–0.34) (*Table 3*) and when accounting for missing outcome data (see Supplementary data online, *Figure S2* and Supplementary data online, *Table S4*). Reductions were seen across stages of AKI (Stage 1: 8.3% vs. 28.0%; Stage 2: 0.7% vs. 2.1%; and Stage 3: 0% vs. 0.4%).

**Table 3:** Outcomes of patients enrolled in the NITRATE-CIN study

	Treatment group		Unadjusted		Covariate adjusted^a^	
	Placebo	Inorganic nitrate				
CIN	*n* = 282	*n* = 274	Odds Ratio (95% CI)	*P* value	Odds Ratio (95% CI)	*P* value
**CIN *n* (%)**	86 (30.50)	25 (9.12)	0.23 (0.14 to 0.37)	<.001	0.21 (0.13 to 0.34)	<.001
Creatinine *(mmol/L)*	112.7 (49.6)	110.1 (43.0)				
eGFR (mL/min/1.73 m^2^)	59.6 (21.1)	60.3 (20.8)				

3 month	*n* = 215	*n* = 206	Difference (95% CI)	*P* value	Difference (95% CI)	*P* value
*Creatinine (mmol/L) Mean ± SD*						
Baseline	97.3 (32.2)	107.3 (38.3)				
3-month	109.6 (41.8)	108.8 (41.5)	−0.74 (−8.72 to 7.24)	.855	−10.42 (−14.98 to −5.86)	<.001
Change	12.3 (25.8)	1.5 (20.9)				

3 month	*n* = 210	*n* = 205	Difference (95% CI)	*P* value	Difference (95% CI)	*P* value
*eGFR Mean ± SD (mL/min/1.73 m^2^)*	*n = 210*	*n = 205*				
Baseline	66.4 (18.1)	60.6 (18.1)				
3-month	59.9 (18.5)	60.5 (17.8)	0.62 (−2.89 to 4.12)	.730	5.17 (2.94 to 7.39)	<.001
Change	−6.58 (12.9)	−0.12 (11.1)				

1 Year MACE	*n* = 320	*n* = 318	Odds Ratio (95% CI)	*P* value	Odds Ratio (95% CI)	*P* value
MACE	58 (18.13)	29 (9.12)	0.45 (0.28 to 0.73)	.001	0.45 (0.28 to 0.73)	.001
All-cause mortality	35 (10.94)	17 (5.35)	0.46 (0.25 to 0.84)	.011	0.46 (0.25 to 0.84)	.011
Cardiovascular mortality	12 (3.75)	8 (2.52)	0.66 (0.27 to 1.64)	.374	0.66 (0.27 to 1.64)	.375
Non-fatal MI	25 (7.81)	10 (3.14)	0.38 (0.18 to 0.81)	.031	0.38 (0.18 to 0.81)	.012
Unscheduled revascularisation	15 (4.69)	5 (1.57)	0.32 (0.12 to 0.90)	.031	0.32 (0.12 to 0.90)	.031

1 year MAKE	*n* = 320	*n* = 318	Odds Ratio (95% CI)	*P* value	Odds Ratio (95% CI)	*P* value
MAKE	91 (28.44)	34 (10.69)	0.30 (0.20 to 0.46)	<.001	0.30 (0.19 to 0.46)	<.001
All-cause mortality	35 (10.94)	17 (5.35)	0.46 (0.25 to 0.84)	.011	0.46 (0.25 to 0.84)	.011
New-onset RRT	5 (1.56)	3 (0.94)	0.60 (0.14 to 2.53)	.487	0.60 (0.14 to 2.55)	.490
Persistent Renal Dysfunction	62 (19.38)	18 (5.66)	0.25 (0.14 To 0.43)	<.001	0.25 (0.14 To 0.43)	<.001

Kidney function at 3 months and 1-year Major adverse cardiac events (MACE) [excluding procedural mis] and Major adverse kidney events (MAKE) are listed. Data is shown as odds ratio with 95% confidence interval (CI) or mean value ± standard deviation (SD) or a number (percentage). MI, Myocardial Infarction; RRT, Renal replacement therapy.

^a^Adjusted differences are corrected for diabetes status with 3-month renal function measures also adjusting for baseline measures.

#### Subgroup analysis

Higher rates of CIN were evident in patients with diabetes (24.9%) vs. non-diabetics (15.6%) and in those with high/very high Mehran scores (25.9%) vs. low/medium Mehran scores (15.6%). Positive benefits of inorganic nitrate therapy on the incidence of CIN were seen irrespective of diabetes status, troponin status, or different Mehran risk group status (see Supplementary data online, *Table S2* and also *Tables S3* and *S4* for analysis accounting for missing data). However, there is evidence of a potential reduction in the effect of inorganic nitrate treatment in patients already receiving organic nitrate (interaction term in logistic regression model: *P* = .040; in patients receiving organic nitrate: odds ratio 0.65, 95% CI 0.20–2.08; in patients with no prior organic nitrate use: odds ratio 0.17, 95% CI 0.10–0.29) (see Supplementary data online, *Table S2*).

We assessed whether reduction in CIN was consistent when using the CIN criteria as per the Mehran model (≥25% or ≥0.5 mg/dL increase in creatinine concentration at 48 h). Using the Mehran model, the overall incidence of CIN (15.8%) was lower than that reported with KDIGO (20%) however the incidence of CIN in those receiving inorganic nitrate remained significantly attenuated [7.3% (18/274)] compared to the placebo group [24.8% (70/282), *P* < .0001].

### Secondary endpoints

#### Tolerance and effect of intervention on plasma nitrate/nitrite levels

Inorganic nitrate intervention and placebo were both tolerated well, with a summary of the adverse and serious adverse events given in the Supplementary data online, *Appendix* (see Supplementary data online, *Figures S2* and *S3* and *Tables S5* and *S6*). There was a significant increase in the plasma nitrate concentration at both 4–6 h (349.5 ± 193.2 μmol/L vs. 34.0 ± 22.4 μmol/L, *P* < .001) and 48–72 h (376.5 ± 260.7 μmol/L vs. 32.2 ± 19.5 μmol/L, *P* < .001) after angiography in those receiving inorganic nitrate when compared to placebo (see Supplementary data online, *Figure S5*). There were also increased levels in plasma nitrite concentrations seen at both 4–6 h (1.40 ± 5.3 μmol/L vs. 0.5 ± 0.37 μmol/L, *P* = .038) and 48–72 h (0.9 ± 1.2 μmol/L vs. 0.4 ± 0.3 μmol/L, *P* = .036) in those receiving inorganic nitrate compared to placebo indicating that the enterosalivary circuit of nitrate was intact in these patients. In contrast, there were no differences in the plasma nitrate (31.5 ± 16.2 μmol/L vs. 33.6 ± 18.6 μmol/L, *P* = .9982) or nitrite concentrations (0.3 ± 0.2 μmol/L vs. 0.3 ± 0.2 μmol/L, *P* = .999) at 3 months between the two groups indicating washout of nitrate in those receiving inorganic nitrate. In addition, assessment of blood pressure confirmed both delivery and efficacy of inorganic nitrate with a reduction in systolic blood pressure and no significant difference between the groups in diastolic blood pressure or heart rate (HR) (see Supplementary data online, *Figure S6*).

#### Three-month kidney function

**Figure 2:**
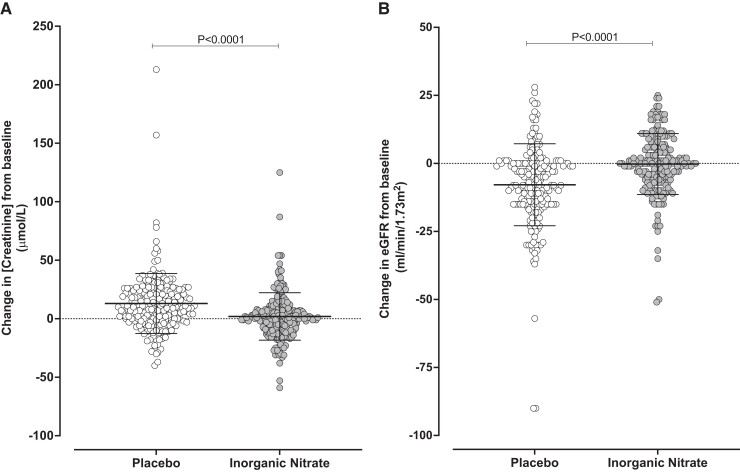
Kidney function is improved at 3 months in patients receiving inorganic nitrate. Panel A shows the change in serum creatinine concentration at 3 months from baseline with a significant decrease in the inorganic nitrate group compared to the placebo group (−10.42 (95% confidence interval: −14.98 to −5.86), *P* < .001; difference as estimated by linear regression adjusting for baseline creatinine and diabetes status). Panel B shows the change in eGFR concentration at 3 months from baseline with a significant increase in the inorganic nitrate group compared to the placebo group (5.17 (95% confidence interval 2.94–7.39), *P* < .001; difference as estimated by linear regression adjusting for baseline creatinine and diabetes status)

Kidney function measures were available for 421 patients at 3 months. There was no difference in the level of creatinine (109.5 ± 41.8 vs. 108.8 ± 41.5 μmol/L, *P* = .855) or level of eGFR (59.9 ± 18.5 vs. 60.5 ± 17.8 mL/min, *P* = .730) between the placebo and inorganic nitrate groups respectively. However, assessment of the change in creatinine or eGFR from baseline to 3 months demonstrated that there was a significant improvement in both measures with inorganic nitrate therapy compared to placebo. At 3 months, a significant increase in creatinine [10.42 μmol/L (95% CI 5.86–14.98), *P* < .001] and a decline in eGFR [5.17 mL/min (95% CI 2.94–7.39), *P* < .001] were evident in the placebo group when compared to those receiving inorganic nitrate (*Table 3* and *Figure 2*). These effects were accompanied by significantly higher rates of persistent kidney dysfunction (20.3% in the placebo group vs. 6.0% in the inorganic nitrate group at 3 months; *P* < .001).

### Outcomes

#### Procedural complications

Rates of procedural MI were significantly reduced in the inorganic nitrate group [2.7% (4 of 147 patients with PCI)] compared to placebo [12.5% (20/160)] (*P* = .003) (see Supplementary data online, *Table S6*).

#### One-year major adverse cardiac events

**Figure 3:**
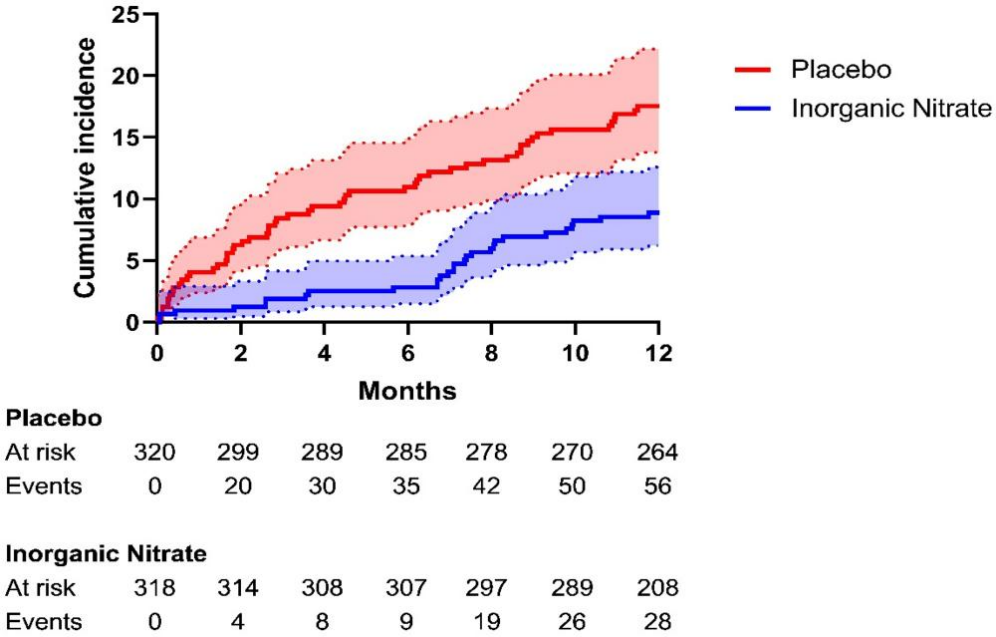
Major adverse cardiac events at 12 months. The cumulative incidence (% of population) of major adverse cardiac events during the 12-month follow-up period was estimated by the Kaplan–Meier method. Two additional patients in the placebo arm and one in the inorganic nitrate arm experienced a major adverse cardiac events event between 12 and 13 months. These events happened within the 1-year follow-up window (12 month ± 30 days)

At 1-year overall MACE rates were 13.6% (87/638). There was a significant reduction in MACE rates in the inorganic nitrate group (9.1%) compared to the placebo group (18.1%, *P* = .001) over the 1-year period. This was driven by reduced rates of all-cause mortality (5.4% vs. 10.9%, *P* = .011), non-fatal MI (3.8% vs. 7.8%, *P* = .033), and unscheduled revascularization (1.6% vs. 4.7%, *P* = .031) (*Figure 3*).

#### One-year major adverse kidney events

**Figure 4:**
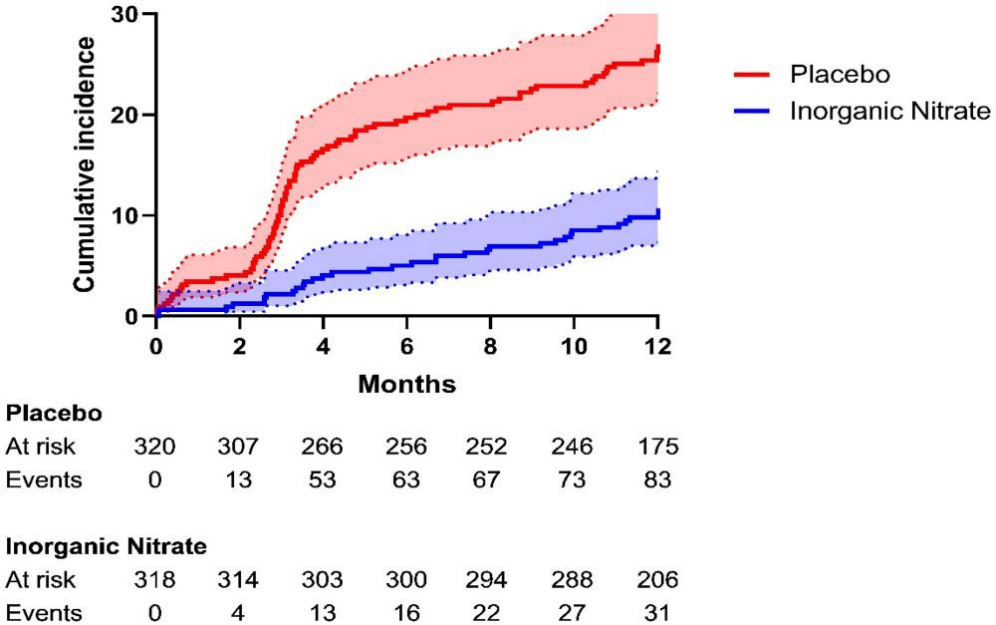
Major adverse kidney events at 12 months. The cumulative incidence (% of population) of major adverse kidney events during the 12-month follow-up period was estimated by the Kaplan–Meier method. Eight additional patients in the placebo arm and three in the Inorganic nitrate arm experienced a major adverse kidney events event between 12 and 13 months. These events happened within the 1-year follow-up window (12 month ± 30 days)

At 1 year, overall MAKE rates were 20.2% (129/638). There was a significant reduction in MAKE rates in the inorganic nitrate group (11.0%) compared to the placebo group (29.4%, *P* < .001) over the 1-year period. This was driven by reduced rates of all-cause mortality (5.4% vs. 10.9%, *P* = .011; full detail in supplement Supplementary data online, *Table S7*) and persistent kidney dysfunction (6.0% vs. 20.3%, *P* < .001) (*Figure 4*).

## Discussion

In this randomized, double-blind, placebo-controlled trial of patients with ACS at risk of CIN undergoing invasive coronary angiography, a short (5 day) once daily treatment with oral inorganic nitrate, initiated before angiography, led to a significant reduction in CIN meeting the primary endpoint of the study. This reduction was observed consistently across levels of CIN risk, diabetic status, and ACS subgroups (NSTEMI/unstable angina). Importantly, this reduction in CIN was also associated with improved kidney outcomes at 3 months and a reduction in both kidney and cardiac events over the following 12 months (*Structured Graphical Abstract*). These findings together support the concept of ‘NO’ replacement in the form of inorganic nitrate as a potential solution to prevent CIN and improve both cardiovascular and kidney outcomes after ACS.

The prognostic significance of CIN after coronary angiography has been debated extensively in recent years.^1–3^ However, there is a growing consensus that CIN (CA-AKI), particularly in patients with pre-existing kidney dysfunction, is prognostically important. This importance has been shown to be relevant for patients with low (e.g. KDIGO stage 1) to severe CIN (CA-AKI), with respect to outcomes.^27^ The reduced occurrence of CIN coupled with the reduction in 1-year MACE with inorganic nitrate treatment supports this previously asserted link. Confidence in the reduced MACE observation is provided by evidence of improvement in multiple other related and connected variables and outcomes. Indeed, there were also reductions in procedural MI and improved kidney outcomes at both 3 and 12 months. In addition to MACE, there was a reduction of all-cause mortality too. Interestingly separation out of this data hints at reductions in infection and stroke-associated mortality in addition to cardiovascular deaths, although due to the low numbers of each individually, a much larger phase 3 study is needed to assess this further.

The demographics of the NITRATE-CIN cohort exposed a relatively high number of NSTE-ACS patients with diabetes and in particular Type II diabetes. It is of note that approximately 25% of the patients recruited were of Asian and African-Caribbean descent; a fact driven by the diversity of the local community that is served by The Barts Heart Centre. It is generally accepted that there is a higher incidence, particularly of Type II diabetes, in those of Asian and African-Caribbean descent. This likely in part underlies the high rates of CIN within the cohort. Recent evidence interrogating the UK Biobank cohort dataset has demonstrated that UK Asians have the highest proportion of Type II diabetes in the UK at 17.86%. Perhaps of greater relevance to NITRATE-CIN is that further analysis of this group demonstrated a 30% prevalence of Type II diabetes in Bangladeshi people; the highest of all the Asian groups analysed within the dataset.^28^ The Barts Heart Centre, as mentioned, serves a very diverse community situated within the East End of London and this part of London is home to a very large Asian (44.4%) and particularly Bangladeshi (34.6% in the 2021 census) community.

Analysis of the plasma indicated a clear rise in circulating nitrate and nitrite levels post-angiography in those receiving the KNO_3_. The rise in nitrite level was commensurate with the level measured in the NITRITE-AMI trial where reductions of CIN were apparent in exploratory assessments,^17^ and thus supporting the assumptions made regarding dose selection in the protocol. The findings also confirm that the enterosalivary circuit of nitrate is intact, in these patients, and that the non-canonical pathway for NO delivery is an efficacious approach to deliver NO in ACS. This is of importance since the bioconversion of nitrate to nitrite occurs within the oral cavity and is due to the metabolic activity of commensal oral bacteria.^29^ Numerous studies have suggested that atherosclerotic disease is associated with alterations in the host gut microbiome,^30^ however if such changes occurred in the oral cavity this did not impact the efficacy of inorganic nitrate. Further confirmation of efficacious delivery of NO emanates from the lowering of systolic blood pressure evident at 4–6 h following IMP administration in comparison to baseline or placebo. This fits well with the known pharmacokinetics of oral inorganic nitrate ingestion.^14^ Both nitrate and nitrite circulate in the blood and are found sequestered in tissues,^31,32^ and represent a significant stable intravascular endocrine reservoir and tissue storage form of NO that exerts beneficial effects.^29,33^ Importantly, this reservoir can be supplemented through exogenous administration of the anion^29^ and our results in this study intimate that the intervention has worked as expected and likely raised tissue concentrations in addition to blood concentrations of both anions. This fact is particularly pertinent to patients at high risk of CIN due to pre-existing CKD, since this latter condition is associated with increases in the levels of endogenous inhibitors of conventional NO synthesis thought to underlie in part the deficient NO state of such patients.^34^

The protective effect of inorganic nitrate against CIN evidenced herein is supported by studies suggesting renoprotective effects of inorganic nitrate or nitrite in pre-clinical studies. Reduction of nitrite to NO following topical^35^ administration of sodium nitrite (30 nmol) protected rats against kidney ischaemia/reperfusion-induced injury *in vivo*, an observation supported by similar studies in mice^36^ and mimicked with dietary inorganic treatment.^37^ Additionally, the protection mechanistically related to NO delivery is supported by clinical data demonstrating some benefits with the direct NO donor isosorbide dinitrate (ISDN) against CIN. In a study of 394 patients, ISDN administration together with hydration reduced CIN rates, in patients with heart failure^38^ as also did administration of organic nitrate in a small study of 199 patients specifically undergoing angioplasty, demonstrating that only 15.2% developed kidney impairment with organic nitrate treatment compared to 29.9% in those who did not.^39^ Further larger studies have yet to be performed to confirm the observations but a key issue limiting the therapeutic potential of organic nitrates is that continuous/repeated administration is of limited benefit due to the development of tolerance.^40^ This issue is not a limiting factor with inorganic nitrate, since there is no evidence of tolerance with either inorganic nitrite or nitrate treatment.^18^ Further support for benefits against CIN, of NO delivery in the ACS setting, comes from a trial showing that inhaled NO reduced the incidence of postoperative acute kidney injury and improved long-term kidney outcomes after cardiopulmonary bypass.^41^ Whilst these data support the concept of CIN reduction and improved outcomes with NO supplementation, the major cost and logistical issues associated with inhaled NO therapy, however, reduce enthusiasm for such an approach. In contrast, treatment with a nitrate salt such as KNO_3_ is simple-to-administer and likely substantially superior as a cost-effective approach to CIN reduction.

Interestingly, in NITRATE-CIN prior use of an organic nitrate appeared to eliminate the positive effects of inorganic nitrate. One could speculate that the exposure to organic nitrate had already recovered NO levels and thus no further benefit was achievable. However, only 72 patients in total received organic nitrate before angiography indicating that there was likely insufficient power for this analysis. It is also worth noting that despite the lack of statistical significance there was a trend for less CIN with inorganic nitrate treatment. Further study powered to detect differences is needed to better assess the impact of organic nitrate on the efficacy of inorganic nitrate.

### Limitations

NITRATE-CIN was a single-centre study, so whether the results will be replicated in patients with different demographics and across other centres with different practices is uncertain. In the baseline demographics there is a trend, although not statistically significant, to slightly more contrast volume and radiation given to patients in the placebo group vs. the inorganic nitrate-treated group. Since the patients were randomized and the study double-blind, we can only conclude that this occurred at random. The study is also powered off rates of CIN and therefore the reduced clinical events to 12 months with inorganic nitrate should be considered as hypothesis-generating and preliminary in nature. Although planned, the cost-effectiveness evaluation of inorganic nitrate in this setting has not been completed so no conclusions on this can be made currently.

## Conclusion

In patients at risk of kidney injury undergoing coronary angiography for ACS, dietary inorganic nitrate reduces CIN compared to placebo. This corresponded to improved kidney outcomes at 3 months and MACE events at 12 months, findings which could have important implications for reducing the burden on the NHS. Further studies powered off MACE events are needed to confirm these findings.

## Supplementary data

Supplementary data are available at *European Heart Journal* online.

## Supplementary Material

ehae100_Supplementary_Data

## Declarations

### Data Availability

Data is available on request to the corresponding author.

### Pre-registered Clinical Trial Number

The pre-registered clinical trial number is ClinicalTrials.gov: NCT03627130.

## References

[1] Mehran R, Dangas GD, Weisbord SD. Contrast-Associated acute kidney injury. N Engl J Med 2019;380:2146–55. 10.1056/NEJMra1805256 31141635

[2] Roger R, Robert MB, Derek JH. Contrast-induced nephropathy following angiography and cardiac interventions. Heart 2016;102:638–48. 10.1136/heartjnl-2014-306962 26857214 PMC4819627

[3] McCullough PA, Choi JP, Feghali GA, Schussler JM, Stoler RM, Vallabahn RC, et al Contrast-Induced acute kidney injury. J Am Coll Cardiol 2016;68:1465–73. 10.1016/j.jacc.2016.05.099 27659469

[4] Azzalini L, Kalra S. Contrast-Induced acute kidney injury-definitions, epidemiology, and implications. Interv Cardiol Clin 2020;9:299–309. 10.1016/j.iccl.2020.02.001 32471671

[5] Kooiman J, Pasha SM, Zondag W, Sijpkens YWJ, van der Molen AJ, Huisman MV, et al Meta-analysis: serum creatinine changes following contrast enhanced CT imaging. Eur J Radiol 2012;81:2554–61. 10.1016/j.ejrad.2011.11.020 22177326

[6] Mohebi R, Karimi Galougahi K, Garcia JJ, Horst J, Ben-Yehuda O, Radhakrishnan J, et al Long-term clinical impact of contrast-associated acute kidney injury following PCI: an ADAPT-DES substudy. JACC Cardiovasc Interv 2022;15:753–66. 10.1016/j.jcin.2021.11.026 35305904

[7] Arrivi A, Truscelli G, Pucci G, Barillà F, Carnevale R, Nocella C, et al The combined treatment of glutathione sodium salt and ascorbic acid for preventing contrast-associated acute kidney injury in ST-elevation myocardial infarction patients undergoing primary PCI: a hypothesis to be validated. Antioxidants 2023;12:773. 10.3390/antiox12030773. 36979021 PMC10045886

[8] Heyman SN, Rosen S, Khamaisi M, Idée J-M, Rosenberger C. Reactive oxygen Species and the pathogenesis of radiocontrast-induced nephropathy. Invest Radiol 2010;45:188–95. 10.1097/RLI.0b013e3181d2eed8 20195159

[9] Arrivi A, Pucci G, Dominici M, Mangieri E, Tanzilli G. Contrast-induced acute kidney injury and nitric oxide depletion in patients undergoing primary percutaneous coronary intervention: insights from GSH 2014 trial. J Cardiovasc Med 2021;22:788–9. 10.2459/JCM.0000000000001154 33399348

[10] Lin H-H, Lee T-S, Lin S-J, Yeh Y-C, Lu T-M, Hsu C-P. DDAH-2 alleviates contrast medium iopromide-induced acute kidney injury through nitric oxide synthase. Clin Sci 2019;133:2361–78. 10.1042/CS20190455 31763675

[11] Liu ZZ, Schmerbach K, Lu Y, Perlewitz A, Nikitina T, Cantow K, et al Iodinated contrast media cause direct tubular cell damage, leading to oxidative stress, low nitric oxide, and impairment of tubuloglomerular feedback. Am J Physiol Renal Physiol 2014;306:F864–72. 10.1152/ajprenal.00302.2013 24431205 PMC4422341

[12] Marenzi G, Assanelli E, Marana I, Lauri G, Campodonico J, Grazi M, et al N-Acetylcysteine and contrast-induced nephropathy in primary angioplasty. N Engl J Med 2006;354:2773–82. 10.1056/NEJMoa054209 16807414

[13] Sandilands EA, Rees JMB, Raja K, Dhaun N, Morrison EE, Hickson K, et al Acetylcysteine has no mechanistic effect in patients at risk of contrast-induced nephropathy: a failure of academic clinical science. Clin Pharmacol Ther 2022;111:1222–38. 10.1002/cpt.2541 35098531 PMC9306485

[14] Kapil V, Milsom AB, Okorie M, Maleki-Toyserkani S, Akram F, Rehman F, et al Inorganic nitrate supplementation lowers blood pressure in humans: role for nitrite-derived NO. Hypertension 2010;56:274–81. 10.1161/HYPERTENSIONAHA.110.153536 20585108

[15] Beirne A-M, Mitchelmore O, Palma S, Andiapen M, Rathod KS, Hammond V, et al NITRATE-CIN Study: protocol of a randomized (1:1) single-center, UK, double-blind placebo-controlled trial testing the effect of inorganic nitrate on contrast-induced nephropathy in patients undergoing coronary angiography for acute coronary syndromes. J Cardiovasc Pharmacol Ther 2021;26:303–9. 10.1177/1074248421000520 33764198 PMC8132002

[16] Jones DA, Khambata RS, Andiapen M, Rathod KS, Mathur A, Ahluwalia A. Intracoronary nitrite suppresses the inflammatory response following primary percutaneous coronary intervention. Heart 2016;103:508–16. 10.1136/heartjnl-2016-309748 27683405

[17] Jones DA, Pellaton C, Velmurugan S, Andiapen M, Antoniou S, van Eijl S, et al Randomized phase 2 trial of intra-coronary nitrite during acute myocardial infarction. Circ Res 2015;116:437–47. 10.1161/CIRCRESAHA.116.305082 25512434 PMC4340586

[18] Kapil V, Khambata RS, Robertson A, Caulfield MJ, Ahluwalia A. Dietary nitrate provides sustained blood pressure lowering in hypertensive patients: a randomized phase 2, double-blind, placebo-controlled study. Hypertension 2015;65:320–7. 10.1161/HYPERTENSIONAHA.114.04675 25421976 PMC4288952

[19] Kidney Disease: Improving Global Outcomes (KDIGO) Acute Kidney Injury Work Group . KDIGO clinical practice guideline for acute kidney injury. Kidney Int Suppl 2012:1–138. https://kdigo.org/wp-content/uploads/2016/10/KDIGO-AKI-Suppl-Appendices-A-F_March2012.pdf

[20] Moussa ID, Klein LW, Shah B, Mehran R, Mack MJ, Brilakis ES, et al Consideration of a new definition of clinically relevant myocardial infarction after coronary revascularization: an expert consensus document from the society for cardiovascular angiography and interventions (SCAI). J Am Coll Cardiol 2013;62:1563–70. 10.1016/j.jacc.2013.08.720 24135581 PMC3890321

[21] Billings FT IV, Shaw AD. Clinical trial endpoints in acute kidney injury. Nephron Clin Pract 2014;127:89–93. 10.1159/000363725 25343828 PMC4480222

[22] Kenneth FS, Douglas GA, David M. CONSORT 2010 statement: updated guidelines for reporting parallel group randomised trials. BMJ 2010;340:c332. 10.1136/bmj.c332 20332509 PMC2844940

[23] Donahoe SM, Stewart GC, McCabe CH, Mohanavelu S, Murphy SA, Cannon CP, et al Diabetes and mortality following acute coronary syndromes. JAMA 2007;298:765–75. 10.1001/jama.298.7.765 17699010

[24] Franklin K, Goldberg RJ, Spencer F, Klein W, Budaj A, Brieger D, et al Implications of diabetes in patients with acute coronary syndromes: the global registry of acute coronary events. Arch Intern Med 2004;164:1457–63. 10.1001/archinte.164.13.1457 15249356

[25] Marenzi G, Cabiati A, Assanelli E. Chronic kidney disease in acute coronary syndromes. World J Nephrol 2012;1:134–45. 10.5527/wjn.v1.i5.134 24175251 PMC3782212

[26] Fox KA, Dabbous OH, Goldberg RJ, Pieper KS, Eagle KA, Van de Werf F, et al Prediction of risk of death and myocardial infarction in the six months after presentation with acute coronary syndrome: prospective multinational observational study (GRACE). BMJ 2006;333:1091. 10.1136/bmj.38985.646481.55 17032691 PMC1661748

[27] Odutayo A, Wong CX, Farkouh M, Altman DG, Hopewell S, Emdin CA, et al AKI and long-term risk for cardiovascular events and mortality. J Am Soc Nephrol 2017;28:377–87. 10.1681/ASN.2016010105 27297949 PMC5198285

[28] Nagar SD, Nápoles AM, Jordan IK, Mariño-Ramírez L. Socioeconomic deprivation and genetic ancestry interact to modify type 2 diabetes ethnic disparities in the United Kingdom. eClinicalMedicine 2021;37:100960. 10.1016/j.eclinm.2021.100960 34386746 PMC8343245

[29] Kapil V, Khambata RS, Jones DA, Rathod K, Primus C, Massimo G, et al The noncanonical pathway for in vivo nitric oxide generation: the nitrate-nitrite-nitric oxide pathway. Pharmacol Rev 2020;72:692–766. 10.1124/pr.120.019240 32576603

[30] Jie Z, Xia H, Zhong S-L, Feng Q, Li S, Liang S, et al The gut microbiome in atherosclerotic cardiovascular disease. Nat Commun 2017;8:845. 10.1038/s41467-017-00900-1 29018189 PMC5635030

[31] Bryan NS, Fernandez BO, Bauer SM, Garcia-Saura MF, Milsom AB, Rassaf T, et al Nitrite is a signaling molecule and regulator of gene expression in mammalian tissues. Nat Chem Biol 2005;1:290–7. 10.1038/nchembio734 16408059

[32] Zweier JL, Wang P, Samouilov A, Kuppusamy P. Enzyme-independent formation of nitric oxide in biological tissues. Nat Med 1995;1:804–9. 10.1038/nm0895-804 7585184

[33] Lundberg JO, Weitzberg E. Nitric oxide signaling in health and disease. Cell 2022;185:2853–78. 10.1016/j.cell.2022.06.010 35931019

[34] Oliva-Damaso E, Oliva-Damaso N, Rodriguez-Esparragon F, Payan J, Baamonde-Laborda E, Gonzalez-Cabrera F, et al Asymmetric (ADMA) and symmetric (SDMA) dimethylarginines in chronic kidney disease: a clinical approach. Int J Mol Sci 2019;20:3668. 10.3390/ijms20153668 31357472 PMC6696355

[35] Tripatara P, Patel NS, Webb A, Rathod K, Lecomte FM, Mazzon E, et al Nitrite-Derived nitric oxide protects the rat kidney against ischemia/reperfusion injury in vivo: role for Xanthine oxidoreductase. J Am Soc.Nephrol 2007;18:570–80. 10.1681/ASN.2006050450 17202421

[36] Milsom AB, Patel NS, Mazzon E, Tripatara P, Storey A, Mota-Filipe H, et al Role for endothelial nitric oxide synthase in nitrite-induced protection against renal ischemia-reperfusion injury in mice. Nitric Oxide 2010;22:141–8. 10.1016/j.niox.2009.10.010 19892029

[37] Zhang G, Han H, Zhuge Z, Dong F, Jiang S, Wang W, et al Renovascular effects of inorganic nitrate following ischemia-reperfusion of the kidney. Redox Biol 2021;39:101836. 10.1016/j.redox.2020.101836 33360353 PMC7772560

[38] Qian G, Liu C-F, Guo J, Dong W, Wang J, Chen Y. Prevention of contrast-induced nephropathy by adequate hydration combined with isosorbide dinitrate for patients with renal insufficiency and congestive heart failure. Clin Cardiol 2019;42:21–5. 10.1002/clc.23023 30054906 PMC6436482

[39] Peguero JG, Cornielle V, Gomez SI, Issa OM, Heimowitz TB, Santana O, et al The use of nitrates in the prevention of contrast-induced nephropathy in patients hospitalized after undergoing percutaneous coronary intervention. J Cardiovasc Pharmacol Ther 2013;19:310–4. 10.1177/1074248413515077 24367008

[40] Münzel T, Steven S, Daiber A. Organic nitrates: update on mechanisms underlying vasodilation, tolerance and endothelial dysfunction. Vascul pharmacol 2014;63:105–13. 10.1016/j.vph.2014.09.002 25446162

[41] Lei C, Berra L, Rezoagli E, Yu B, Dong H, Yu S, et al Nitric oxide decreases acute kidney injury and stage 3 chronic kidney disease after cardiac surgery. Am J Respir Crit Care Med 2018;198:1279–87. 10.1164/rccm.201710-2150OC 29932345 PMC6290943

