# Supplementary material for: Inorganic nitrate benefits contrast-induced nephropathy after coronary angiography for acute coronary syndromes: the NITRATE-CIN trial
Source: Eur Heart J. 2024 Mar 21;45(18):1647–58. doi: 10.1093/eurheartj/ehae100 (PMC11089333; doi:10.1093/eurheartj/ehae100)
Supplement: ehae100_Supplementary_Data [file ehae100_supplementary_data.docx]

**Supplemental Material: Inorganic nitrate benefits contrast-induced nephropathy after coronary angiography for acute coronary syndromes: the NITRATE-CIN trial**

**Contents**

**1. NITRATE-CIN Investigators, Committees and Collaborators**

**2. KDIGO Criteria for the primary endpoint**

**3. Table S1. Baseline demographics of the trial population split by ethnicity.**

**4. Figure S1. Contrast Amount by treatment group in the NITRATE-CIN cohort.**

**5. Table S2. Subgroup Analysis of the primary endpoint (CIN rates).**

**6. Table S3. Baseline Characteristics for patients with and without missing primary outcome (CIN) data.**

**7. Figure S2. Results from the sensitivity analysis examining the effect of missingness in the primary outcome.**

**8. Table S4. Key results from the sensitivity analysis examining the effect of missingness in the primary outcome.**

**9. Figure S3. Adverse Events by System Organ Class and Treatment allocation.**

**10. Table S5. Adverse Events by Treatment allocation.**

**11. Figure S4. Serious adverse Events by System Organ Class and Treatment allocation.**

**12. Table S6. Serious adverse Events by Treatment allocation.**

**13. Figure S5. Plasma Nitrate/Nitrite levels over time in the NITRATE-CIN cohort.**

**14. Figure S6. Blood pressure and heart rate haemodynamics in the NITRATE-CIN cohort.**

**15. Table S7. Cause of death by treatment group**

**1. NITRATE-CIN Investigators, Committees and Collaborators**

**Investigators:** Dr Daniel Jones, Dr Anne-Marie Beirne, Dr Matthew Kelham, Dr Krishnaraj S. Rathod, Ms Tipparat Parakaw, Mervyn Andiapen, Lucinda Wynne, Mrs Jessica Adams, Miss Anna Learoyd, Prof Magdi Yaqoob, Prof Anthony Mathur, Prof Amrita Ahluwalia

**Trial Steering Committee:** Dr Andrew Wragg, Dr Rajiv Amersey, Dr Suzanne Forbes, Mr Paul Wright

**Data Safety and Monitoring Board:** Dr Rob Bell, Dr Alex Sirker, Mr Sotiris Antoniou, Dr Oliver Guttmann, Professor Rhian Gabe

**CVCTU:** Marian Benford, Shahana Chowdhury, Victoria Hammond, Simon Menezes

**2. KDIGO Criteria**

The primary incidence of CIN was assessed using the Kidney Disease Improving Global Outcomes (KDIGO) clinical practice guidelines for acute kidney injury (AKI)^1^

KDIGO defines AKI as any of the following:

- Increase in serum creatinine by 0.3mg/dL or more within 48 hours **or**
- Increase in serum creatinine to 1.5 times baseline or more within the last 7 days **or**
- Urine output less than 0.5 mL/kg/hr for 6 hours

| **AKI Stage** | **Serum Creatinine (SCr) criteria** | **Urine output criteria** |
| --- | --- | --- |
| 1 | SCr increase ＞26μmol/L within 48h  or  SCr increase ＞1.5-2x reference SCr within 1 week | ＜0.5mL/kg/h for 6 consecutive hours |
| 2 | SCr increase ＞2-3x reference SCr within 1 week | ＜0.5mL/kg/h for 12h |
| 3 | SCr increase ＞3x reference SCr within 1 week  or  SCr increase ＞354μmol/L  or  Initiated on RRT (irrespective of stage at time of initiation) | ＜0.5mL/kg/h for 24h  or  anuria for 12h |

|  | **Asian**  **(N=103)** | **Black**  **(N=54)** | **White**  **(N=481)** | **P-value** |
| --- | --- | --- | --- | --- |
| **Age (years), mean (SD)** | 63.84 (11.83) | 67.47 (10.91) | 72.17 (11.45) | <0.001 |
| **Female, n (%)** | 18 (17%) | 21 (40%) | 132 (27%) | 0.012 |
| **BMI (kg/m^2^), mean (SD)** | 27.77 (5.14) | 30.17 (6.37) | 28.33 (5.69) | 0.027 |
| **NSTEMI, n (%)** | 85 (81%) | 48 (89%) | 410 (85%) | 0.589 |
| **eGFR <60mls/min, n (%)** | 42 (40.8%) | 27 (50%) | 223 (46.6%) | 0.474 |
| **Diabetes, n (%)** | 70 (67%) | 34 (62%) | 190 (40%) | <0.001 |
| **CIN, n (%)** | 17 (18%) | 9 (18%) | 85 (20%) | 0.872 |

**Table S1. Baseline demographics of the trial population split by ethnicity.** This highlights the proportion of patients qualifying for CIN prophylaxis in each criterion according to ethnicity. There were 2 patients of Oriental ethnicity who are not included due to small sample size. Groups are compared using One-Way Anova for continuous and chi squared for categorical outcomes.

**Figure S1. Contrast Amount by treatment group in the NITRATE-CIN cohort.** Scatter plot showing the contrast amounts administered during angiography by treatment group. Values shown are mean± SD.

|  |  |  | **Treatment group** | | **Covariate adjusted*** |  |
| --- | --- | --- | --- | --- | --- | --- |
|  | Subgroup | **All patients**  **N=556** | Placebo  N=282 | Inorganic Nitrate  N=274 | OR (95% CI) | Interaction P value |
| **CIN, number (%)** | Pre-existing organic nitrate use, N=72 | 16 (22.22) | 10 (24.39) | 6 (19.35) | 0.65 (0.20 to 2.08) | 0.040 |
|  | No prior organic nitrate use, N=484 | 95 (19.63) | 76 (31.54) | 19 (7.82) | 0.17 (0.10 to 0.29) |  |
|  | Diabetic, N=261 | 65 (24.90) | 49 (36.30) | 16 (12.70) | 0.23 (0.12 to 0.45) | 0.583 |
|  | Non-diabetic, N=295 | 46 (15.59) | 37 (25.17) | 9 (6.08) | 0.18 (0.08 to 0.39) |  |
|  | Troponin positive, N=480 | 95 (19.79) | 74 (30.45) | 21 (8.86) | 0.20 (0.12 to 0.35) | 0.837 |
|  | Troponin negative, N=76 | 16 (21.05) | 12 (30.77) | 4 (10.81) | 0.24 (0.07 to 0.83) |  |
|  | Mehran risk score – (≤10), N=320 | 50 (15.6) | 43 (24.43) | 7 (4.86) | 0.16 (0.07 to 0.36) | 0.520 |
|  | Mehran risk score – (≥11), N=236 | 61 (25.85) | 43 (40.57) | 18 (13.85) | 0.22 (0.12 to 0.42) |  |

**Table S2**. **Subgroup Analysis of the primary endpoint (CIN rates).** Pre-existing organic nitrate use is defined as Isosorbide mononitrate (ISMN) or dinitrate (ISDN) or intravenous glyceryl trinitrate (GTN). Diabetes included all types (type 1 (1.9%), type II diet controlled (12.6%), type II drug therapy (63.2%) and type II insulin controlled (22.2%)). Analysis is adjusted for baseline creatinine and diabetes status (unless this is the interaction of interest). OR = Odds ratio, CI = Confidence Interval

**Sensitivity analysis for missing primary outcome data**

Methods

The main analysis was completed under the assumption that missing data in the primary outcome (in 84 patients) was missing completely at random. A pattern mixture model was implemented examining the robustness of this assumption using the logistic regression model adjusting for diabetes status and creatinine level at baseline. The informative missingness odds ratio (IMOR) was set at a baseline of 1 (no relationship between the CIN outcome and missingness) and varied between 0 and 2 at intervals of 0.2 for either both treatment arms, the placebo arm only or the inorganic nitrate arm only. One patient with missing CIN data was also missing baseline creatinine data. In line with the SAP, this patient was excluded from the pattern mixture model.

Examination of the baseline characteristics of patients with missing primary outcome data (Table S3) indicated that some baseline characteristics were related to missingness. To account for this scenario, a second pattern mixture model using the same parameters as indicated above was completed with the addition of the following auxillary variables: age, prior PCI, presentation, and LV ejection fraction. A binary indicator of LV impairment was not included (despite significance when comparing groups in Table S3) due to its relatedness with LV ejection fraction. 14 patients were missing LV ejection fraction data. The mean LV ejection fraction was imputed for these patients with a missingness indicator included as an additional covariate.

Results

The pattern mixture model set to the baseline IMOR of 1 for both arms provided the expected results for the logistics regression model i.e. odds ratio 0.21 (95% CI 0.13 to 0.34). This varied very little when the IMOR was adjusted in both arms (Table S4, Figure S2a).

When the IMOR was adjusted only in the placebo arm, reducing the IMOR slightly reduced the estimated difference in odds of CIN between the two study arms (at IMOR=0 in placebo arm, odds ratio 0.25 (95% CI 0.15 to 0.40); Figure S2a) and increasing the IMOR slightly increased the estimated difference in odds of CIN between the two study arms (at IMOR=2 in placebo arm, odds ratio 0.19 (95% CI 0.12 to 0.31); Figure S2a).

When the IMOR was adjusted only in the inorganic nitrate arm, reducing the IMOR slightly increased the estimated difference in odds of CIN between the two study arms (at IMOR=0 in inorganic nitrate arm, odds ratio 0.18 (95% CI 0.11 to 0.29); Figure S2a) and increasing the IMOR slightly reduced the estimated difference in odds of CIN between the two study arms (at IMOR=2 in inorganic nitrate arm, odds ratio 0.24 (95% CI 0.14 to 0.38); Figure S2a).

Including auxiliary variables potentially predictive of missingness in the outcome had little effect on the obtained odds ratios described above (Table S4, Figure S2b)

|  | | | **Patients included in primary analysis (N=556)** | **Patients with missing CIN data (N=84)** | **Comparison between groups** |
| --- | --- | --- | --- | --- | --- |
| **Age (years), mean ± SD** | | | 71.4 ± 11.4 | 68.4 ± 13.1 | P=0.024 |
| **Sex** | | Female, n (%) | 153 (27.5) | 18 (21.4) | P=0.24 |
|  |  | Male, n (%) | 403 (73.3) | 66 (78.6) |  |
| **Ethnicity** | | Asian, n (%) | 89 (16.0) | 13 (15.5) | P=0.55 |
|  |  | Black, n (%) | 49 (8.8) | 4 (4.8) |  |
|  |  | White, n (%) | 415 (74.6) | 66 (78.6) |  |
|  |  | Unknown, n (%) | 3 (0.5) | 1 (1.2) |  |
| **BMI (kg/m^2^), mean ± SD** | | | 28.2 ± 5.7 | 29.1 ± 5.5 | P=0.16 |
| **Hypertension, n (%)** | | | 424 (76.3) | 61 (72.6) | P=0.47 |
| **Hypercholesterolaemia, n (%)** | | | 190 (59.0) | 196 (61.0) | P=0.75 |
| **Previous PCI, n (%)** | | | 175 (31.5) | 37 (44.0) | P=0.022 |
| **Previous MI, n (%)** | | | 176 (31.7) | 34 (40.5) | P=0.11 |
| **Diabetes, n (%)** | | | 261 (46.9) | 33 (39.3) | P=0.19 |
|  | Type I, n (%) | | 5 (0.9) | 1 (1.2) | P=0.30 |
|  | Type II: Diet controlled, n(%) | | 33 (5.9) | 8 (9.5) |  |
|  | Type II: Drug therapy, n(%) | | 165 (29.7) | 18 (21.4) |  |
|  | Type II: Insulin, n(%) | | 58 (10.4) | 6 (7.1) |  |
| **Presentation** | | Unstable angina, n (%) | 76 (13.7) | 21 (25.0) | P=0.007 |
|  |  | NSTEMI, n (%) | 480 (86.3) | 63 (75.0) |  |
| **Smoking History** | | Non- smoker, n (%) | 236 (42.4) | 32 (38.1) | P=0.29 |
|  |  | Previous smoker, n (%) | 249 (44.8) | 36 (42.9) |  |
|  |  | Current smoker, n (%) | 71 (12.8) | 16 (19.0) |  |
| **Peripheral Vascular Disease, n (%)** | | | 24 (4.3) | 2 (2.4) | P=0.40 |
| **Stroke, n (%)** | | | 32 (5.8) | 6 (7.1) | P=0.62 |
| **LV impairment, n (%)** | |  | 226 (41.6) | 19 (22.9) | P=0.001 |
| **LV Ejection Fraction, mean ± SD** | | | 47.1 ± 12.1 | 51.9 ± 9.1 | P<0.001 |
| **SBP (mmHg), mean ± SD** | | | 131.8 ± 20.5 | 132.6 ± 19.0 | P=0.73 |
| **DBP (mmHg), mean ± SD** | | | 71.7 ± 10.5 | 73.9 ± 11.7 | P=0.088 |
| **HR (bpm), mean ± SD** | | | 70.1 ± 12.6 | 69.1 ± 10.2 | P=0.51 |

**Table S3. Baseline Characteristics for patients with and without missing primary outcome (CIN) data.** Comparisons between groups are made using either t-tests (continuous characteristics) or chi-square tests (categorical characteristics) and informed the choice of auxiliary variables included in the second pattern mixture model. NSTEMI: Non-ST Elevation Myocardial Infarction, PCI: Percutaneous Coronary Intervention.


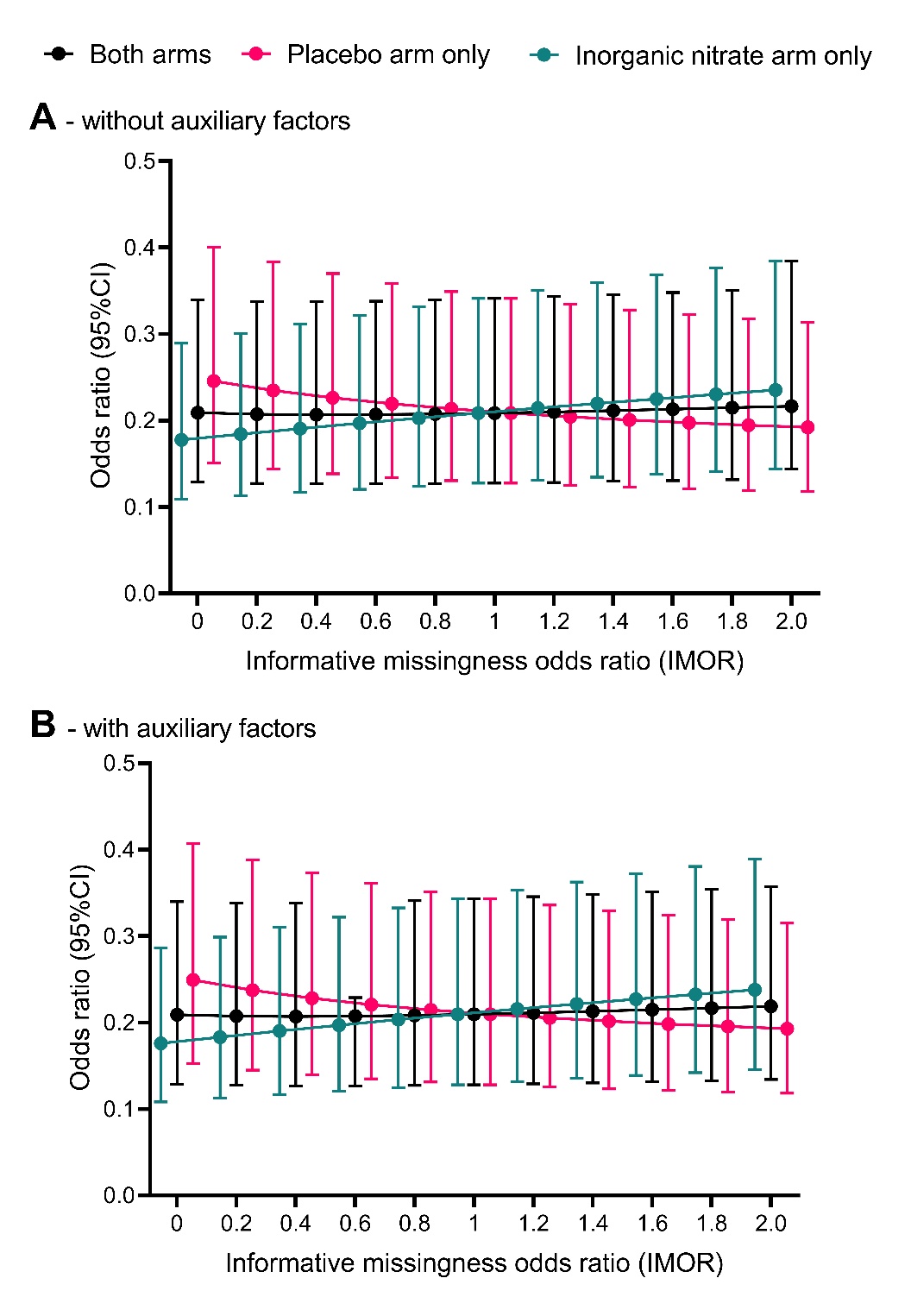


**Figure S2. Results from the sensitivity analysis examining the effect of missingness in the primary outcome.** Informative missingness odds ratio was varied from a baseline of 1 to values ranging from 0 to 2 in both arms (black), the placebo arm only (red), the inorganic nitrate arm only (green). This analysis was completed without any auxiliary factors predicting missingness (A) and with 4 auxiliary factors predicting missingness (B). This had little effect on the obtained odds ratio for the odds of CIN (the primary outcome) in the inorganic nitrate arm vs. the placebo arm. CI: confidence interval.

| IMOR | Change of IMOR in study arm | Odds ratios (95% CI) from | |
| --- | --- | --- | --- |
|  |  | **Pattern mixture model** | **Pattern mixture model with auxiliary variables** |
| 0 | Both arms | 0.21 (0.13 to 0.34) | 0.21 (0.13 to 0.34) |
|  | Placebo arm only | 0.25 (0.15 to 0.41) | 0.25 (0.15 to 0.41) |
|  | Inorganic nitrate arm only | 0.18 (0.11 to 0.29) | 0.18 (0.11 to 0.29) |
| 1 (baseline) | Both arms | 0.21 (0.13 to 0.34) | 0.21 (0.13 to 0.34) |
|  | Placebo arm only | 0.21 (0.13 to 0.34) | 0.21 (0.13 to 0.34) |
|  | Inorganic nitrate arm only | 0.21 (0.13 to 0.34) | 0.21 (0.13 to 0.34) |
| 2 | Both arms | 0.22 (0.13 to 0.35) | 0.22 (0.13 to 0.36) |
|  | Placebo arm only | 0.19 (0.12 to 0.31) | 0.19 (0.12 to 0.31) |
|  | Inorganic nitrate arm only | 0.24 (0.14 to 0.38) | 0.24 (0.15 to 0.39) |

**Table S4. Key results from the sensitivity analysis examining the effect of missingness in the primary outcome.** IMOR: informative missingness odds ratio. CI: Confidence interval.

**NITRATE-CIN: Adverse event data**

There were 184 AEs in 156 patients. More than twice as many patients in the placebo arm experienced AEs (38.1% vs. 16.4%, p<0.001). Most patients experienced 1 AE while 24 patients experienced two AEs (15 patients in the placebo arm vs. 5 in the active arm) and 2 patients experienced three AEs (both on the placebo arm).

Of these AEs 75.5% (139/184) were procedure-related events occurring in 116 patients. 16 patients experienced 2 procedural AEs: 14 in the placebo arm vs. 2 in the active arm. Only 31 procedure-related AEs were myocardial infarctions.

Most AEs were of a mild intensity (83.2%, 153/184). 15.8% of AEs were graded moderate intensity (29/184) while 2 AES were graded as severe (1.09%).


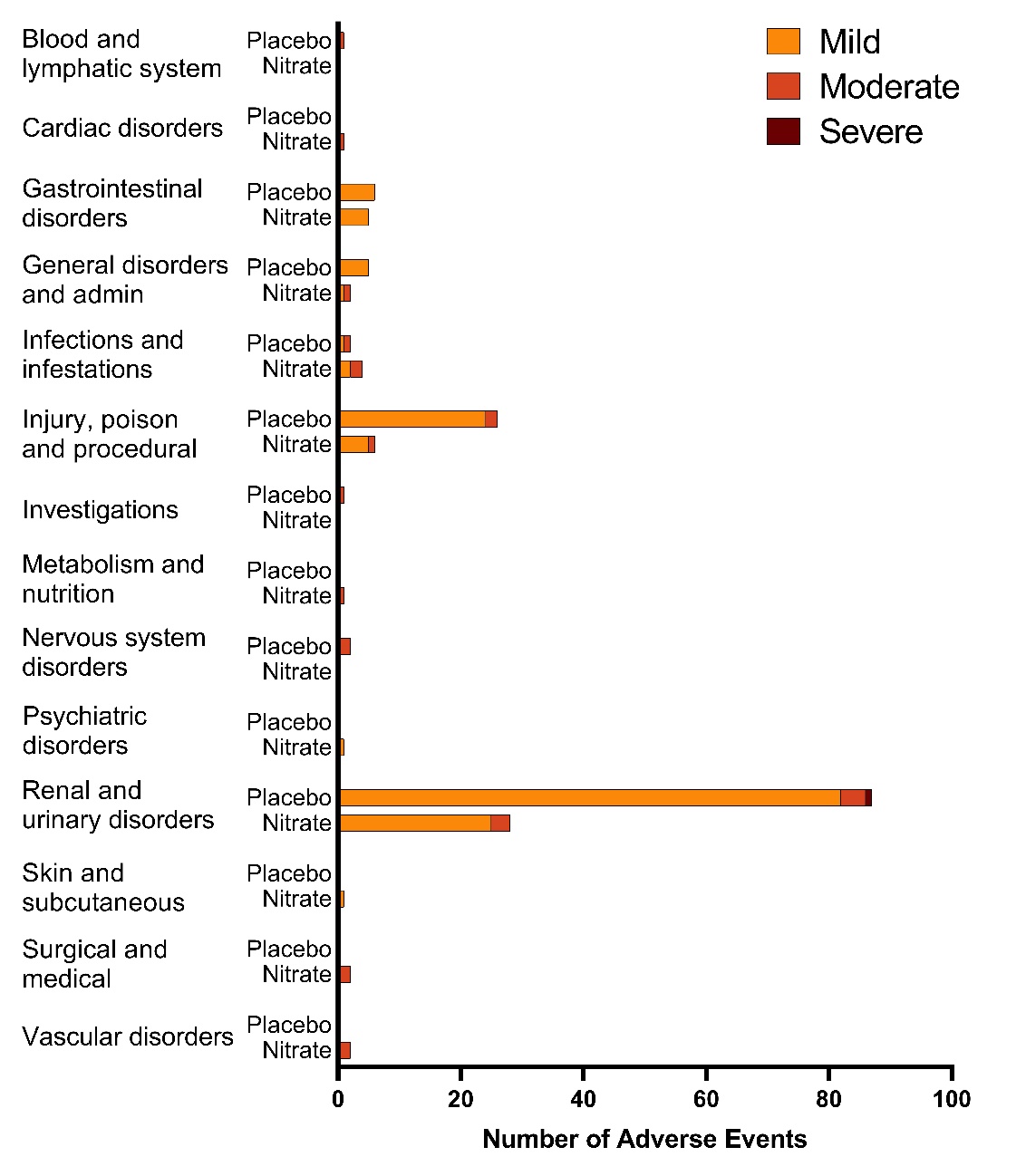


**Figure S3. Adverse Events by System Organ Class and Treatment allocation.** The numbers of events in each applicable system organ class are shown stacked by severity: orange=mild, red=moderate, brown=severe. The most frequent adverse event was acute kidney injury (renal and urinary disorders) followed by procedure-related myocardial infarctions (injury, poisoning and procedural).

|  | **Placebo,**  **N = 321** | | | | **Inorganic Nitrate,**  **N = 319** | | | |
| --- | --- | --- | --- | --- | --- | --- | --- | --- |
| **Adverse Event** | **Grade Mild** | **Grade Moderate** | **Grade Severe** | ***Overall*** | **Grade Mild** | **Grade Moderate** | **Grade Severe** | ***Overall*** |
| **Total** | **118** | **11** | **2** | ***131*** | **40** | **13** | **0** | ***53*** |
| **Blood and lymphatic system disorders** | **—** | **1 (0.5)** | **—** | ***1 (0.5)*** | **—** | **—** | **—** | ***—*** |
| Anaemia | — | 1 (0.5) | — | *1 (0.5)* | — | — | — | *—* |
| **Cardiac disorders** | **—** | **—** | **—** | **—** | — | **1 (0.5)** | **—** | ***1 (0.5)*** |
| Cardiac failure | — | — | — | *—* | — | 1 (0.5) | — | *1 (0.5)* |
| **Gastrointestinal disorders** | **6 (3.3)** | **—** | **—** | ***6 (3.3)*** | **5 (2.7)** | **—** | **—** | ***5 (2.7)*** |
| Gastritis | 6 (3.3) | — | — | *6 (3.3)* | 2 (1.1) | — | — | *2 (1.1)* |
| Nausea | — | — | — | *—* | 3 (1.6) | — | — | *3 (1.6)* |
| **General disorders and administration site conditions** | **5 (2.7)** | **—** | **—** | ***5 (2.7)*** | **1 (0.5)** | **1 (0.5)** | **—** | ***2 (1.1)*** |
| Chest pain | 4 (2.2) | — | — | *4 (2.2)* | 1 (0.5) | 1 (0.5) | — | *2 (1.1)* |
| Malaise | 1 (0.5) | — | — | *1 (0.5)* | — | — | — | *—* |
| **Infections and infestations** | **1 (0.5)** | **1 (0.5)** | **—** | ***1 (0.5)*** | **2 (1.1)** | **2 (1.1)** | **—** | ***4 (2.2)*** |
| SARS-COVID result positive | 1 (0.5) | — | — | *1 (0.5)* | 1 (0.5) | 1 (0.5) | — | *2 (1.1)* |
| Cellulitis | — | 1 (0.5) | — | *1 (0.5)* | — | — | — | *—* |
| Pneumonia | — | — | — | *—* | — | 1 (0.5) | — | *1 (0.5)* |
| Gum Infection | — | — | — | — | 1 (0.5 | — | — | *1 (0.5)* |
| **Injury, poisoning and procedural complications** | **24 (13.0)** | **2 (1.1)** | **—** | ***26 (14.1)*** | **5 (2.7)** | **1 (0.5)** | **—** | ***6 (3.3)*** |
| Fracture | — | 1 (0.5) | — | *1 (0.5)* | — | — | — | *—* |
| Procedure-related MI | 24 (13.0) | 1 (0.5) | — | *25 (13.6)* | 5 (2.7) | 1 (0.5) | — | *6 (3.3)* |
| **Investigations** | **—** | **1 (0.5)** | **—** | ***1 (0.5)*** | **—** | **—** | **—** | ***—*** |
| Angiogram | — | 1 (0.5) | — | *1 (0.5)* | — | — | — | *—* |
| **Metabolism and nutrition disorders** | **—** | **—** | **—** | ***—*** | **—** | **1 (0.5)** | **—** | ***1 (0.5)*** |
| Diabetes mellitus | — | — | — | *—* | — | 1 (0.5) | — | *1 (0.5)* |
| **Nervous system disorders** | **—** | **2 (1.1)** | **—** | ***2 (1.1)*** | **—** | **—** | **—** | ***—*** |
| Presyncope | — | 1 (0.5) | — | *1 (0.5)* | — | — | — | *—* |
| Transient ischaemic attack | — | 1 (0.5) | — | *1 (0.5)* | — | — | — | *—* |
| **Psychiatric disorders** | **—** | **—** | **—** | ***—*** | **1 (0.5)** | **—** | **—** | ***1 (0.5)*** |
| Confusion | — | — | — | *—* | 1 (0.5) | — | — | *1 (0.5)* |
| **Renal and urinary disorders** | **82 (44.3)** | **4 (2.2)** | **1 (0.5)** | ***87 (47.3)*** | **25 (13.6)** | **3 (1.6)** | **—** | ***28 (15.2)*** |
| Acute kidney injury | 82 (44.3) | 4 (2.2) | 1 (0.5) | *87 (47.3)* | 25 (13.6) | 2 (1.1) | — | *27 (14.7)* |
| Chronic kidney disease | — | — | — | — | — | 1 (0.5) | — | *1 (0.5)* |
| **Skin and subcutaneous tissue disorders** | **—** | **—** | **—** | ***—*** | **1 (0.5)** | **—** | **—** | ***1 (0.5)*** |
| Rash | — | — | — | *—* | 1 (0.5) | — | — | *1 (0.5)* |
| **Surgical and medical procedures** | **—** | **—** | **1 (0.5)** | ***1 (0.5)*** | **—** | **2 (1.1)** | **—** | ***2 (1.1)*** |
| Cardiac resynchronisation therapy | — | — | — | *—* | — | 1 (0.5) | — | *1 (0.5)* |
| Coronary artery bypass | — | — | — | *—* | — | 1 (0.5) | — | *1 (0.5)* |
| Renal replacement therapy | — | — | 1 (0.5) | *1 (0.5)* | — | — | — | *—* |
| **Vascular disorders** | **—** | **—** | **—** | ***—*** | **—** | **2 (1.1)** | **—** | ***2 (1.1)*** |
| Haematoma | — | — | — | *—* | — | 1 (0.5) | — | *1 (0.5)* |
| Hypotension | — | — | — | *—* | — | 1 (0.5) | — | *1 (0.5)* |

**Table S5. Adverse Events by Treatment allocation.** Numbers are presented with % of total adverse events (n=184). The total number of adverse events in each organ class are highlighted in bold followed by a breakdown within that class. The total number of adverse events experienced in each trial arm are highlighted in italics.

**NITRATE-CIN: Serious adverse event data**

There were 340 SAEs in 220 patients. Similar numbers of patient experienced SAEs in both trial arms (36.1% in placebo vs. 32.6% in active, p=0.346). Most patients experienced 1 SAE although the number experienced by each patient varied from 1 to 9 (range in active arm: 1-5, range in placebo arm: 1-9). 68 patients experienced multiple SAEs with equal numbers in the active (n=35) and placebo (n=33) arms.

Of these SAEs 82.4% (280/340) resulted in hospitalisation or prolongation of hospitalisation. These SAEs were equally divided between trial arms (140 in each arm). 52 SAEs resulted in patient death with the majority of fatality SAEs occurring in the placebo arm (35 in placebo vs. 17 in active). Of the remaining SAEs, 7 were life-threatening and 1 was an important medical procedure (renal replacement therapy).

Most SAEs were of a moderate intensity (68.5%, 233/340). 22.1% of SAEs were graded severe (75/340) whilst the remaining 9.4% were graded as mild (32/340).


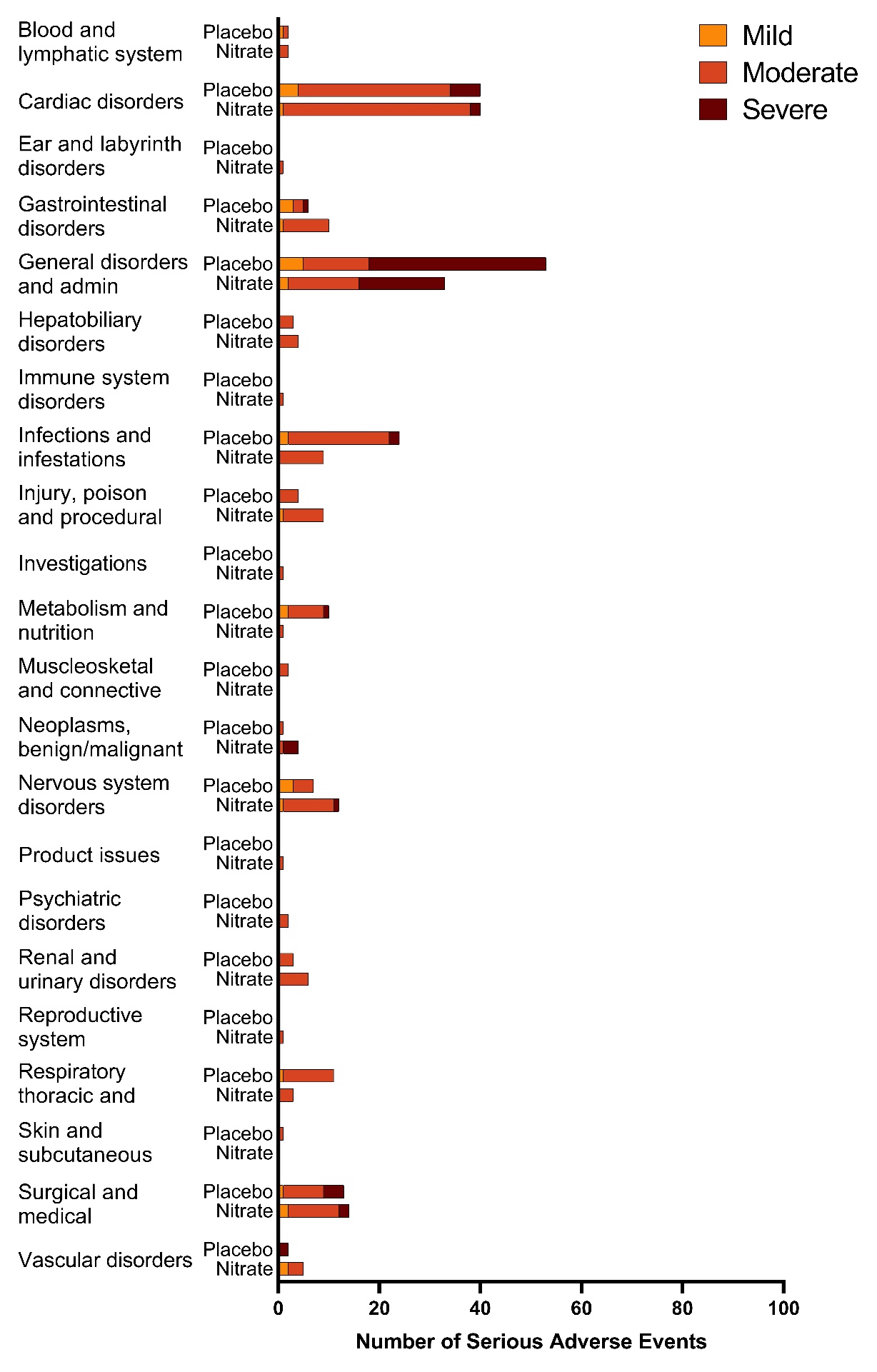


**Figure S4. Serious adverse Events by System Organ Class and Treatment allocation.** The numbers of events in each system organ class are shown stacked by severity: orange=mild, red=moderate, brown=severe. The most frequent serious adverse events were death (general disorders and admin) and myocardial infarctions (cardiac disorders).

|  | **Placebo,**  **N = 321** | | | | **Inorganic Nitrate,**  **N = 319** | | | |
| --- | --- | --- | --- | --- | --- | --- | --- | --- |
| **Serious Adverse Event** | **Grade Mild** | **Grade Moderate** | **Grade Severe** | ***Overall*** | **Grade Mild** | **Grade Moderate** | **Grade Severe** | ***Overall*** |
| **Total** | **22** | **109** | **50** | ***181*** | **10** | **124** | **25** | ***159*** |
| **Blood and lymphatic system disorders** | **1 (0.3)** | **1 (0.3)** | **—** | ***2 (0.6)*** | **—** | **2 (0.6)** | **—** | ***2 (0.6)*** |
| Anaemia | 1 (0.3) | 1 (0.3) | — | *2 (0.6)* | — | 1 (0.3) | — | *1 (0.3)* |
| Thrombocytopenia | — | — | — | — | — | 1 (0.3) | — | *1 (0.3)* |
| **Cardiac disorders** | **4 (1.2)** | **30 (8.8)** | **6 (1.8)** | ***40 (11.8)*** | **1 (0.3)** | **37 (10.9)** | **2 (0.6)** | ***40 (11.8)*** |
| Acute coronary syndrome | — | — | — | — | — | 1 (0.3) | — | *1 (0.3)* |
| Angina | 1 (0.3) | 2 (0.6) | — | *3 (0.9)* | 1 (0.3) | 6 (1.8) | — | *7 (2.1)* |
| Atrial fibrillation | — | 6 (1.8) | — | *6 (1.8)* | — | 6 (1.8) | — | *6 (1.8)* |
| Atrioventricular block | — | — | — | — | — | 1 (0.3) | — | *1 (0.3)* |
| Bradycardia | — | — | — | — | — | 1 (0.3) | — | *1 (0.3)* |
| Cardiac failure | — | 5 (1.5) | 2 (0.6) | *7 (2.1)* | — | 8 (2.4) | — | *8 (2.4)* |
| Myocardial infarction | 3 (0.9) | 17 (5.0) | 4 (0.6) | *24 (7.1)** | — | 8 (2.4) | 2 (0.6) | *10 (2.9)* |
| Pericardial effusion | — | — | — | — | — | 1 (0.3) | — | *1 (0.3)* |
| Ventricular arrhythmia | — | — | — | — | — | 5 (1.5) | — | *5 (1.5)* |
| **Ear and labyrinth disorders** | **—** | **—** | **—** | **—** | **—** | **1 (0.3)** | **—** | ***1 (0.3)*** |
| Vertigo | — | — | — | — | — | 1 (0.3) | — | *1 (0.3)* |
| **Gastrointestinal disorders** | **3 (0.9)** | **2 (0.6)** | **1 (0.3)** | ***6 (1.8)*** | **1 (0.3)** | **9 (2.6)** | **—** | ***10 (2.9)*** |
| Abdominal pain | — | 1 (0.3) | — | *1 (0.3)* | — | 2 (0.6) | — | *2 (0.6)* |
| Divericultitis | — | 1 (0.3) | — | *1 (0.3)* | — | 1 (0.3) | — | *1 (0.3)* |
| Gastritis | 1 (0.3) | — | — | *1 (0.3)* | 1 (0.3) | 2 (0.6) | — | *3 (0.9)* |
| Peptic ulcer haemorrhage | — | — | — | — | — | 1 (0.3) | — | *1 (0.3)* |
| Rectal bleed | — | — | 1 (0.3) | *1 (0.3)* | — | 2 (0.6) | — | *2 (0.6)* |
| Upper GI bleed | 1 (0.3) | — | — | *1 (0.3)* | — | 1 (0.3) | — | *1 (0.3)* |
| Vomiting | 1 (0.3) | — | — | *1 (0.3)* | — | — | — | — |
| **General disorders administration site conditions** | **5 (1.5)** | **13 (3.8)** | **35 (10.3)** | ***53 (15.6)*** | **2 (0.6)** | **14 (4.1)** | **17 (5.0)** | ***33 (9.7)*** |
| Chest pain | 4 (1.2) | 6 (1.8) | — | *10 (2.9)* | 2 (0.6) | 12 (3.5) | — | *14 (4.1)* |
| Death | — | — | 35 (10.3) | *35 (10.3)* | — | — | 17 (5.0) | *17 (5.0)* |
| Fluid overload | — | — | — | — | — | 1 (0.3) | — | *1 (0.3)* |
| Light headedness | — | — | — | — | — | 1 (0.3) | — | *1 (0.3)* |
| Pyrexia | — | 1 (0.3) | — | *1 (0.3)* | — | — | — | — |
| Shortness of breath | 1 (0.3) | 4 (1.2) | — | *5 (1.5)* | — | — | — | — |
| Swelling | — | 2 (0.6) | — | *2 (0.6)* | — | — | — | — |
| **Hepatobiliary disorders** | **—** | **3 (0.9** | **—** | ***3 (0.9)*** | **—** | **4 (1.2)** | **—** | ***4 (1.2)*** |
| Cholecystitis | — | 1 (0.3) | — | *1 (0.3)* | — | 3 (0.9) | — | *3 (0.9)* |
| Cholelithiasis | — | 2 (0.6) | — | *2 (0.6)* | — | 1 (0.3) | — | *1 (0.3)* |
| **Immune system disorders** | **—** | **—** | **—** | **—** | **—** | **1 (0.3)** | **—** | ***1 (0.3)*** |
| Amyloidosis | — | — | — | — | — | 1 (0.3) | — | *1 (0.3)* |
| **Infections and infestations** | **2 (0.6)** | **20 (5.9)** | **2 (0.6)** | ***24 (7.1)*** | **—** | **9 (2.6)** | **—** | ***9 (2.6)*** |
| Appendicitis | — | — | — | — | — | 1 (0.3) | — | *1 (0.3)* |
| Cellulitis | — | — | — | — | — | 1 (0.3) | — | *1 (0.3)* |
| COVID-19 pneumonia | — | 3 (0.9) | — | *3 (0.9)* | — | — | — | — |
| Cystitis | — | — | — | — | — | 1 (0.3) | — | *1 (0.3)* |
| Pneumonia | 1 (0.3) | 2 (0.6) | 1 (0.3) | *4 (1.2)* | — | 3 (0.9) | — | *3 (0.9)* |
| Respiratory tract infection | — | 9 (2.6) | — | *9 (2.6)* | — | 1 (0.3) | — | *1 (0.3)* |
| Sepsis | — | 1 (0.3) | — | *1 (0.3)* | — | — | — | — |
| Urinary tract infection | 1 (0.3) | 4 (1.2) | — | *5 (1.5)* | — | 1 (0.3) | — | *1 (0.3)* |
| Prosthetic valve endocarditis | — | — | 1 (0.3) | *1 (0.3)* | — | — | — | — |
| Infection, source unknown | — | — | — | — | — | 1 (0.3) | — | *1 (0.3)* |
| **Injury, poisoning and procedural complications** | **—** | **4 (1.2)** | **—** | ***4 (1.2)*** | **1 (0.3)** | **8 (2.4)** | **—** | ***9 (2.6)*** |
| Fall | — | 2 (0.6) | — | *2 (0.6)* | 1 (0.3) | 4 (1.2) | — | *5 (1.5)* |
| Fracture | — | 1 (0.3) | — | *1 (0.3)* | — | — | — | — |
| Subdural haematoma | — | — | — | — | — | 3 (0.9) | — | *3 (0.9)* |
| Subdural haemorrhage | — | — | — | — | — | 1 (0.3) | — | *1 (0.3)* |
| Toxicity to various agents | — | 1 (0.3) | — | *1 (0.3)* | — | — | — | — |
| **Investigations** | **—** | **—** | **—** | **—** | **—** | **1 (0.3)** | **—** | ***1 (0.3)*** |
| Urethroscopy | — | — | — | — | — | 1 (0.3) | — | *1 (0.3)* |
| **Metabolism and nutrition** | **2 (0.6)** | **7 (2.1)** | **1 (0.3)** | ***10 (2.9)*** | **—** | **1 (0.3)** | **—** | ***1 (0.3)*** |
| Dehydration | 1 (0.3) | — | — | *1 (0.3)* | — | — | — | — |
| Diabetic ketoacidosis | — | 2 (0.6) | — | *2 (0.6)* | — | — | — | — |
| Hyperglycaemia | 1 (0.3) | 2 (0.6) | — | *3 (0.9)* | — | — | — | — |
| Hyperkalaemia | — | 2 (0.6) | — | *2 (0.6)* | — | — | — | — |
| Hypoglycaemia | — | — | 1 (0.3) | *1 (0.3)* | — | 1 (0.3) | — | *1 (0.3)* |
| Hyponatraemia | — | 1 (0.3) | — | *1 (0.3)* | — | — | — | — |
| **Musculoskeletal and connective** | **—** | **2 (0.6)** | **—** | ***2 (0.6)*** | **—** | **—** | **—** | **—** |
| Pain | — | 2 (0.6) | — | *2 (0.6)* | — | — | — | — |
| **Neoplasms benign, malignant** | **—** | **1 (0.3)** | **—** | ***1 (0.3)*** | **—** | **1 (0.3)** | **3 (0.9)** | ***4 (1.2)*** |
| Breast cancer | — | — | — | — | — | — | 1 (0.3) | *1 (0.3)* |
| Lung cancer | — | — | — | — | — | — | 1 (0.3) | *1 (0.3)* |
| Metastatic cancer | — | — | — | — | — | 1 (0.3) | 1 (0.3) | *2 (0.6)* |
| Pancreatic cancer | — | 1 (0.3) | — | *1 (0.3)* | — | — | — | — |
| **Nervous system disorders** | **3 (0.9)** | **4 (1.2)** | **—** | ***7 (2.1)*** | **1 (0.3)** | **10 (2.9)** | **1 (0.3)** | ***12 (3.5)*** |
| Cerebral haemorrhage | — | — | — | — | — | 2 (0.6) | — | *2 (0.6)* |
| Cerebrovascular accident | — | 2 (0.6) | — | *2 (0.6)* | — | 4 (1.2) | 1 (0.3) | *5 (1.5)* |
| Dizziness | 2 (0.6) | — | — | *2 (0.6)* | 1 (0.3) | — | — | *1 (0.3)* |
| Loss of consciousness | 1 (0.3) | 1 (0.3) | — | *2 (0.6)* | — | 2 (0.6) | — | *2 (0.6)* |
| Transient ischaemic attack | — | 1 (0.3) | — | *1 (0.3)* | — | 2 (0.6) | — | *2 (0.6)* |
| **Product issues** | **—** | **—** | **—** | **—** | **—** | **1 (0.3)** | **—** | ***1 (0.3)*** |
| Device occlusion | — | — | — | — | — | 1 (0.3) | — | *1 (0.3)* |
| **Psychiatric disorders** | **—** | **—** | **—** | **—** | **—** | **2 (0.6)** | **—** | ***2 (0.6)*** |
| Confusion | — | — | — | — | — | 1 (0.3) | — | *1 (0.3)* |
| Delirium | — | — | — | — | — | 1 (0.3) | — | *1 (0.3)* |
| **Renal and urinary disorders** | **—** | **3 (0.9)** | **—** | ***3 (0.9)*** | **—** | **6 (1.8)** | **—** | ***6 (1.8)*** |
| Acute kidney injury | — | 2 (0.6) | — | *2 (0.6)* | — | 2 (0.6) | — | *2 (0.6)* |
| Haematuria | — | — | — | — | — | 1 (0.3) | — | *1 (0.3)* |
| Renal failure | — | — | — | — | — | 1 (0.3) | — | *1 (0.3)* |
| Urinary retention | — | 1 (0.3) | — | *1 (0.3)* | — | 2 (0.6) | — | *2 (0.6)* |
| **Reproductive system and breast** | **—** | **—** | **—** | **—** | **—** | **1 (0.3)** | **—** | ***1 (0.3)*** |
| Benign prostatic hyperplasia | — | — | — | — | — | 1 (0.3) | — | *1 (0.3)* |
| **Respiratory, thoracic and mediastinal disorders** | **1 (0.3)** | **10 (2.9)** | **—** | ***11 (3.2)*** | **—** | **3 (0.9)** | **—** | ***3 (0.9)*** |
| COPD | — | 1 (0.3) | — | *1 (0.3)* | — | 2 (0.6) | — | *2 (0.6)* |
| Epistaxis | — | 5 (1.5) | — | *5 (1.5)* | — | — | — | — |
| Pleural Effusion | 1 (0.3) | 2 (0.6) | — | *3 (0.9)* | — | 1 (0.3) | — | *1 (0.3)* |
| Pulmonary oedema | — | 2 (0.6) | — | *2 (0.6)* | — | — | — | — |
| **Skin and subcutaneous tissue disorders** | **—** | **1 (0.3)** | **—** | ***1 (0.3)*** | **—** | **—** | **—** | **—** |
| Diabetic foot | — | 1 (0.3) | — | *1 (0.3)* | — | — | — | — |
| **Surgical and medical procedures** | **1 (0.3)** | **8 (2.4)** | **4 (1.2)** | ***13 (3.8)*** | **2 (0.6)** | **10 (2.9)** | **2 (0.6)** | ***14 (4.1)*** |
| Coronary artery bypass | — | 1 (0.3) | — | *1 (0.3)* | — | 1 (0.3) | — | *1 (0.3)* |
| Chemotherapy | — | — | — | — | — | 1 (0.3) | — | *1 (0.3)* |
| Hip replacement | — | 1 (0.3) | — | *1 (0.3)* | — | 1 (0.3) | — | *1 (0.3)* |
| Hospitalisation, unknown cause | — | 3 (0.9) | — | *3 (0.9)* | 1 (0.3) | 1 (0.3) | — | *2 (0.6)* |
| ICD upgrade | — | — | — | — | — | 1 (0.3) | — | *1 (0.3)* |
| Ileostomy | — | 1 (0.3) | — | *1 (0.3)* | — | — | 1 (0.3) | *1 (0.3)* |
| Knee replacement | — | — | — | — | — | 1 (0.3) | — | *1 (0.3)* |
| Percutaneous coronary intervention | — | — | — | — | 1 (0.3) | 1 (0.3) | — | *2 (0.6)* |
| RATS/Segmentectomy | — | — | — | — | — | 1 (0.3) | — | *1 (0.3)* |
| Renal replacement therapy | — | 1 (0.3) | 3 (0.9) | *4 (1.2)^+^* | — | — | 1 (0.3) | *1 (0.3)^+^* |
| Sternotomy | — | — | 1 (0.3) | *1 (0.3)* | — | — | — | — |
| Transcatheter aortic valve implantation | — | — | — | — | — | 1 (0.3) | — | *1 (0.3)* |
| Transurethral prostatectomy | 1 (0.3) | 1 (0.3) | — | *2 (0.6)* | — | 1 (0.3) | — | *1 (0.3)* |
| **Vascular disorders** | **—** | **—** | **2 (0.6)** | ***2 (0.6)*** | **2 (0.6)** | **3 (0.9)** | **—** | ***5 (1.5)*** |
| Haematoma | — | — | — | *—* | — | 1 (0.3) | — | 1 (0.3) |
| Hypertension | — | — | — | *—* | 1 (0.3) | — | — | 1 (0.3) |
| Hypotension | — | — | 1 (0.3) | *1 (0.3)* | — | 2 (0.6) | — | *2 (0.6)* |
| Peripheral ischaemia | — | — | 1 (0.3) | *1 (0.3)* | 1 (0.3) | — | — | 1 (0.3) |

**Table S6. Serious adverse Events by Treatment allocation.** Numbers are presented with % of total serious adverse events (n=340). The total number of adverse events in each organ class are highlighted in bold followed by a breakdown within that class. The total number of adverse events experienced in each trial arm are highlighted in italics.

*An SAE of myocardial infarction is recorded 24 times in 21 placebo patients. 4 additional patients are recorded as having non-fatal myocardial infarctions in outcome data but do not have corresponding SAEs.

^+^3 additional patients (2 active, 1 placebo) were administered renal replacement therapy shortly before death. SAE is recorded as death.

**Figure S5. Plasma Nitrate/Nitrite levels over time in the NITRATE-CIN cohort.** Figure A shows the concentration of plasma nitrate at 4-6h and 48-72h after IMP administration in the inorganic nitrate (KNO_3_, 12mmol daily for five days) group compared to placebo (KCl 12mmol daily for five days) with no difference in the groups at 3 months. Figure B demonstrates changes in plasma nitrite concentrations at 4-6 h and 48-72h after IMP administration. Values shown are mean± SEM of n=431 at 4-6hrs, 94 at 48-72hrs and 105 at 3 months. Two-way ANOVA shown as a number within the graph with Sidak’s post-test comparing groups at each timepoint shown as ** for P<0.01 or **** for P<0.0001

**Figure S6. Blood pressure and heart rate haemodynamics in the NITRATE-CIN cohort.** Figure shows systolic blood pressure (A), diastolic blood pressure (B) and heart rate (C) at baseline, 4-6h after the first IMP administration and then at 3 months in the inorganic nitrate treated (KNO_3_, 12mmol daily for five days; N=316, 262 and 111 respectively)) group compared to placebo (KCl 12mmol daily for five days; N=319, 263, 106 respectively). Values shown as mean± SEM. Two-way ANOVA shown as a number within the graph with Sidak’s post-test comparing groups at each timepoint shown as *** for P<0.001.

|  | **Placebo**  **320)** | **Inorganic Nitrate**  **(N=318)** |
| --- | --- | --- |
| **All Cause Mortality** | 35 (10.94%) | 17 (5.35%) |
| Stroke | 4 (1.25%) | 1 (0.31%) |
| Malignancy | 4 (1.25%) | 4 (1.26%) |
| Infection (including COVID-19) | 12 (3.75%) | 4 (1.26%) |
| GI Haemorrhage | 1 (0.31%) | 0 (0%) |
| Diabetes Mellitus | 1 (0.31%) | 0 (0%) |
| Pulmonary Embolism | 1 (0.31%) | 0 (0%) |
| **Cardiovascular Mortality** | 12 (3.75%) | 8 (2.52%) |

**Table S7: Breakdown of all-cause mortality into the different causes of death.** Death certificate information was available to verify all causes.

References

1. Kidney Disease: Improving Global Outcomes (KDIGO) Acute Kidney Injury Work Group. KDIGO Clinical Practice Guideline for Acute Kidney Injury. Kidney Int Supplements 2012:2:1-138.
